# IL10-driven STAT3 signalling in senescent macrophages promotes pathological eye angiogenesis

**DOI:** 10.1038/ncomms8847

**Published:** 2015-08-11

**Authors:** Rei Nakamura, Abdoulaye Sene, Andrea Santeford, Abdelaziz Gdoura, Shunsuke Kubota, Nicole Zapata, Rajendra S. Apte

**Affiliations:** 1 Department of Ophthalmology and Visual Sciences, Washington University School of Medicine, 660 South Euclid Avenue, Box 8096, St Louis, MO 63110, USA; 2 Department of Developmental Biology, Washington University School of Medicine, St Louis, MO 63110, USA

## Abstract

Macrophage dysfunction plays a pivotal role during neovascular proliferation in diseases of ageing including cancers, atherosclerosis and blinding eye disease. In the eye, choroidal neovascularization (CNV) causes blindness in patients with age-related macular degeneration (AMD). Here we report that increased IL10, not IL4 or IL13, in senescent eyes activates STAT3 signalling that induces the alternative activation of macrophages and vascular proliferation. Targeted inhibition of both IL10 receptor-mediated signalling and STAT3 activation in macrophages reverses the ageing phenotype. In addition, adoptive transfer of STAT3-deficient macrophages into eyes of old mice significantly reduces the amount of CNV. Systemic and CD163^+^ eye macrophages obtained from AMD patients also demonstrate STAT3 activation. Our studies demonstrate that impaired SOCS3 feedback leads to permissive IL10/STAT3 signalling that promotes alternative macrophage activation and pathological neovascularization. These findings have significant implications for our understanding of the pathobiology of age-associated diseases and may guide targeted immunotherapy.

## Abstract (web summary)

Pathological neovascularization causes blinding eye disease. Here the authors show that IL10 activates STAT3 signalling in the macrophages in the ageing eye, promoting their polarization towards a pro-angiogenic phenotype; interfering with this pathway reverses the pathology in a mouse model.

## Graphical abstract

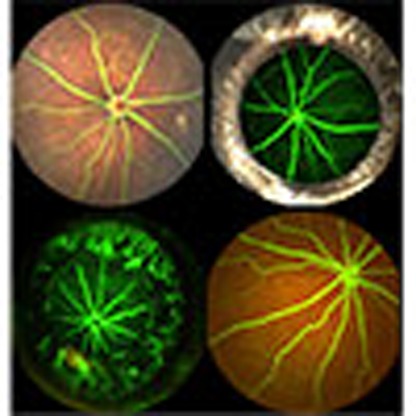

The innate immune system plays an integral role in regulating angiogenesis during development and in age-associated diseases such as cancers, atherosclerosis and blinding eye diseases^1^,^2^,^3^. In the eye, pathological neovascularization during disease progression is observed in patients with age-related macular degeneration (AMD)^4^,^5^. AMD is the leading cause of blindness in people over 50 years of age in the industrialized world and consists of two subsets of disease^6^. Dry (non-neovascular) AMD is characterized by yellowish cholesterol-rich deposits that contain proteolytic fragments of complement factors underneath the retina^7^,^8^,^9^,^10^. Abnormalities in cholesterol metabolism and alternative macrophage activation have been shown to play a significant role in chronic inflammation and disease progression in AMD^5^. Of significant interest is the wet (exudative or neovascular) form of AMD that is associated with the majority of the vision loss. It is characterized by the development of abnormal, leaky blood vessels underneath the retina, a process called choroidal neovascularization (CNV)^11^. Over the last two decades, numerous studies have demonstrated the essential role of innate immunity, especially macrophages, in regulating CNV in AMD^12^,^13^,^14^,^15^. Furthermore, it has become increasingly evident that the polarization state of the activated macrophage determines the pathophysiological fate of the neovascular phenotype^16^,^17^,^18^,^19^. However, the programmatic molecular signals that guide macrophage polarization, particularly during ageing eye diseases, remain unclear^17^,^20^,^21^. The goal of this study was to identify the molecular signalling mechanisms that govern senescent macrophage polarization and inflammation causing neovascular proliferation and blindness in age-associated eye diseases.

Macrophages are incredibly plastic cells that can adapt their activity based on the surrounding micro-environment, confounding the precise understanding of their role in regulating angiogenesis in disease processes^17^,^22^,^23^. There are two dominant states of macrophage activation described: classical activation (M1) or alternative activation (M2). M2 macrophages are further subdivided into M2a and M2c based on the upstream cytokines that drive their induction and function. M2a activation is induced by interleukin (IL)-4/IL13, and it has been recently implicated in the wound-healing process^16^. M2c activation is induced by IL10 and leads to the development of a regulatory macrophage with anti-inflammatory activity^17^. A more recent macrophage classification has been proposed based on their activation conditions^24^,^25^. These new experimental standards further highlight the diversity of macrophage populations. The recognition of multiple polarization states led to the discovery that alternatively activated macrophages, but not M1 macrophages, promoted angiogenesis in tumours and age-related eye disease^14^,^16^,^22^. In addition, macrophages from mice 18 months or older polarize more readily to an alternatively activated phenotype^14^,^26^. Adoptive transfer of M1-like young syngeneic macrophages, but not M2-like old macrophages, into old recipient mice resulted in significant reduction of CNV in a murine model of AMD^14^. Furthermore, a recent study identified that pharmacological inhibition of M2 macrophage polarization by doxycycline specifically decreased CNV^27^. These studies demonstrate the crucial role of alternative polarization of macrophages in promoting CNV in the aged eye.

IL10 is a classic anti-inflammatory cytokine and its molecular signalling pathway has been well characterized in macrophages and T lymphocytes. Secreted IL10 cytokine binds to the IL10 receptor 1 (IL10R1) on membrane surfaces, and IL10R1 dimerizes with IL10R2 to exert its downstream effects. IL10R2 recruits cytoplasmic protein Jak1 followed by phosphorylation of tyrosine at position 705 in the STAT3 (705Y-STAT3) molecule. Phosphorylated STAT3 forms a homodimer, which is then translocated to the nucleus to facilitate transcriptional regulation of target genes such as vascular endothelial growth factor A (VEGF-A), basic fibroblast growth factor-2 and placental growth factor, all major angiogenic factors. Because aberrant angiogenesis is implicated in diverse pathological conditions, multiple therapeutic strategies have aimed at targeting the STAT3 signalling pathway and downstream growth factors. In fact, there are three drugs targeting VEGF-A that are currently approved by the US Food and Drug Administration (FDA) for treating AMD patients with ocular neovascularization. Finally and more importantly, while anti-VEGF-A therapy has been a crucial breakthrough in the ability to treat patients with blinding neovascular eye diseases, the therapeutic regimen is not ideal and has limitations because (1) genetic ablation of *Vegf-a* in macrophages showed no therapeutic benefit in CNV models implicating factors other than VEGF-A in the macrophage-mediated promotion of CNV^16^, (2) treatment requires intraocular injection every few weeks and (3) not every patient responds to the treatment possibly due to genetic variance^28^. Although IL10/IL10R signalling has been shown as a determinant factor in macrophage polarization to an M2 phenotype, the exact mechanism by which ageing transforms them into disease-promoting cells is not well understood. Therefore, elucidating the mechanism by which ageing influences alternative activation of macrophages and how this process contributes to the progression of CNV in blinding eye disease remains imperative.

In this study, we investigated the molecular mechanism behind age-dependent modulation of macrophage polarization and angiogenic function using mice of various age and genetic backgrounds. Here we have shown that IL10 and its downstream STAT3 signalling activity are key regulators of the senescent macrophage phenotype in the eye. We further demonstrate that these age-related modulations in macrophage polarization and function can be rescued by targeting STAT3 activity. Taken together, our study demonstrates the therapeutic potential of targeting STAT3 activity in senescent macrophages as an attractive avenue to restore anti-angiogenic properties of these cells to prevent vision loss. These findings may also be relevant to other disease such as atherosclerosis and cancers where alternatively activated macrophages promote disease pathophysiology.

## Results

### IL10 is elevated in eyes of old mice

**Figure 1:**
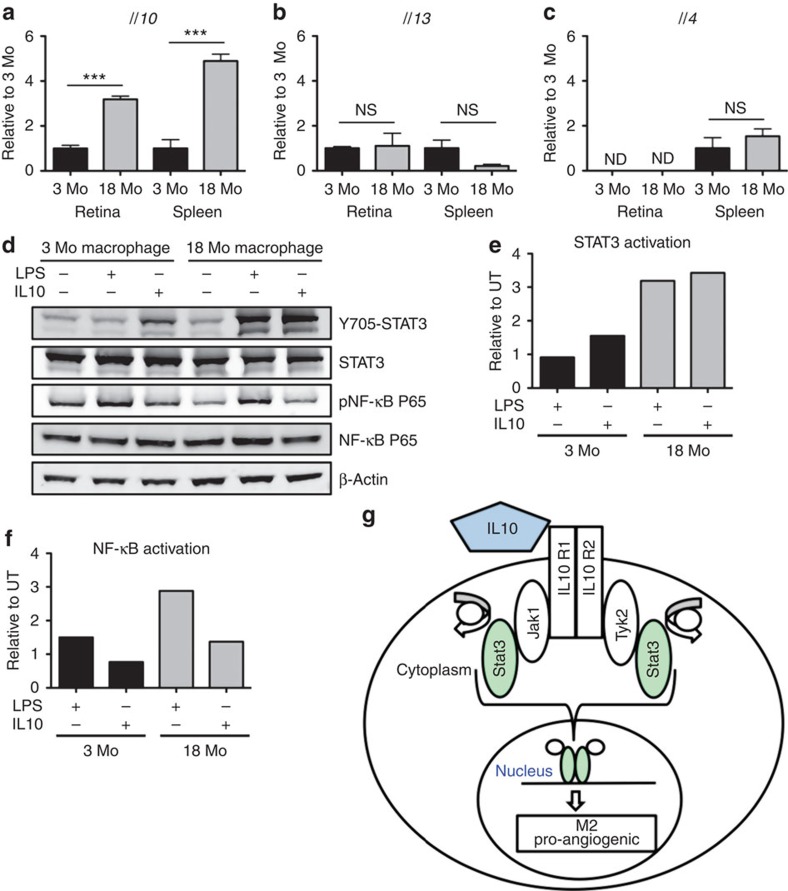
Ageing is associated with elevated IL10 in the eye and increased response to IL10 stimulus in macrophages. (**a**) *Il10*, (**b**) *Il13* and (**c**) *Il4* mRNA expression of retina–choroid and spleen from 3- (3 Mo) and 18-month-old (18 Mo) mice was measured by real-time qPCR. Values are expressed as mean+s.e.m. relative to young (3 Mo) mice. At least three biological replicates were analysed in three independent experiments. (Student's unpaired *t*-test; ***P<0.001, NS (not significant) *P*>0.05 compared with 3-Mo-old mice; ND not detected). (**d**) Peritoneal macrophages from young and old mice were treated with LPS or IL10 for 18 h. Immunoblot with specific antibodies for Y705-STAT3 (79/86 kDa), STAT3 (79/86 kDa), phospho-NF-κB P65 (65 kDa) and NF-κB P65 (65 kDa) subunits were analysed from young and old macrophage whole-cell lysate. These are representative blots from three independent experiments. (**e**) Immunoblot was quantified for the phospho-Y705-STAT3 to total STAT3 ratio and (**f**) for the phospho-P65 to total P65 ratio. The ratios are normalized to the untreated (UT) samples from each age group. (**g**) Proposed model: elevated IL10 and enhanced STAT3 signalling promote alternative activation (M2) in macrophages associated with pro-angiogenic function.

In previous studies, we demonstrated that ageing transformed macrophages from an M1 to an M2 phenotype^14^,^26^. To determine whether these programmatic changes are secondary to upregulation of specific upstream cytokines within the macrophage micro milieu, we analysed the expression levels of M2-polarizing cytokines (*Il4, Il10* and *Il13*) by real-time quantitative PCR (qPCR) in the retina and spleen of young (3 months of age) and old (18 months of age) mice. In the naive state, RNA isolated from the whole retina demonstrated an almost threefold increase in *Il10* expression in old mice versus young mice. In addition, we observed a similar level of upregulation in the spleen, suggesting that the elevated *Il10* expression in old mice is not restricted to the eye, but is rather a systemic phenotype (Fig. 1a and Supplementary Fig. 1a). In contrast, analysis of other M2-polarizing cytokines revealed that ageing had no effects on *Il13* expression in either the eye or spleen, and *Il4* levels were not detectable in the eye (Fig. 1b,c). Therefore, *Il10* was the only M2-polarizing cytokine that was upregulated by ageing in diverse tissues rich in macrophages.

The elevated ocular expression of *Il10* suggested that it could be a critical regulator of macrophage function in age-related eye disease. For instance, IL10 production was increased in the retina of aged mice and the highest levels were associated with areas of massive macrophage infiltration in injury-induced neovascular complexes (Supplementary Fig. 1b). Next, we explored the effect of elevated IL10 on the activation state of macrophages isolated from young and old mice after IL10 or lipopolysaccharides (LPS) stimulation (Fig. 1d). Western blot analysis demonstrated a striking phenotypic difference between the two age groups tested. Old macrophages treated with IL10, as well as LPS, responded via activation of STAT3 signalling at significantly higher amplitude of intensity than young macrophages (Fig. 1d,e). In addition, the nuclear factor-κB (NF-κB) pathway, which primarily responds to LPS treatment but can be activated by IL10, did not show a marked difference between the two age groups (Fig. 1f). Taken altogether, these data demonstrate that ageing increases IL10 expression and enhanced IL10-mediated STAT3 signalling in macrophages (Fig. 1g).

### IL10R deficiency inhibits vascular proliferation

**Figure 2:**
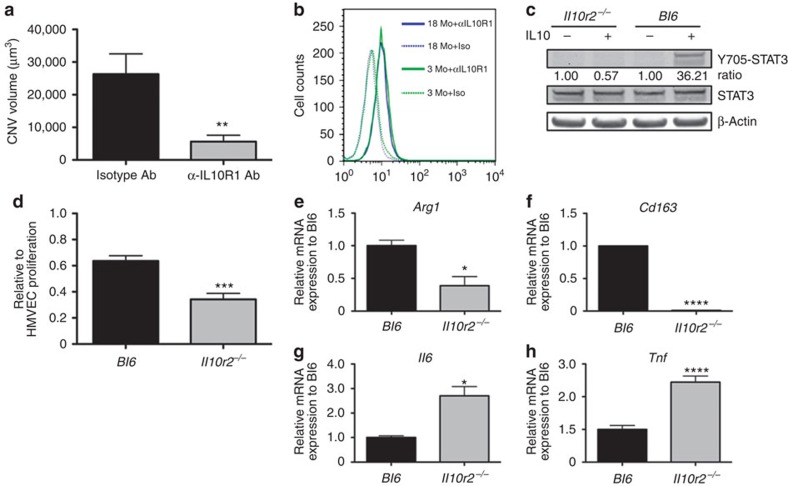
IL10 receptors play a role in regulating CNV, microvascular proliferation and alternative (M2) macrophage polarization. (**a**) Eighteen-month-old female mice were treated with anti-IL10R1 or isotype control antibody (Ab) and CNV complex was quantified. Values are expressed as mean+s.e.m. (*N*>5 eyes per group, Student's unpaired *t*-test; **P<0.01 compared with isotype control). (**b**) Surface IL10R1 expression in young and old peritoneal macrophage was measured using flow cytometry. Macrophages were first gated for F4/80 expression followed by IL10R1 detection using specific antibodies. Representative data from two individual mice for each age group are shown. (**c**) Peritoneal macrophage from C57BL/6J (Bl6) or *Il10r2*^*−/−*^ mice were treated with IL10. Representative blots are shown from peritoneal macrophages treated with recombinant IL10. β-Actin (42 kDa) was used to normalize protein loading, and relative phosphorylated STAT3 (79/86 kDa) signalling was expressed as a ratio to total STAT3 (79/86 kDa). Representative data from three independent experiments are shown. (**d**) HMVEC proliferation assay of *Il10r2*^*−/−*^ macrophage showing enhanced inhibition of HMVEC proliferation compared with age-matched C57BL/6J macrophages. Values are expressed as mean+s.e.m. relative to lone HMVEC cultures with four independent experiments. (*N*>4 co-cultures per group per experiment, Student's unpaired *t*-test; ***P<0.001 compared with C57BL/6J macrophages). mRNA expression of (**e**) *Arg1*, (**f**) *Cd163*, (**g**) *Il6* and (**h**) *Tnf* in peritoneal macrophages from age-matched C57BL/6J and *Il10r2*^*−/−*^ were measured by real-time qPCR. Values are expressed as mean+s.e.m. relative to C57BL/6J (Bl6). Data shown are representative of three independent experiments. *N*>3 biological replicate per group per experiment, Student's unpaired *t*-test; *P<0.05, ***P<0.001 compared with C57BL/6JB macrophages.

To further characterize the potential impact of age-dependent effect of IL10 signalling on macrophage function, we examined the role of the IL10R in regulating macrophage effector functions. To assess this, we first investigated the effect of selectively blocking IL10R-mediated signalling in the injury-induced CNV model, a murine surrogate for neovascularization in AMD. Briefly, old mice (18 months) received intravitreal injections of neutralizing antibody against IL10R1 or an isotype control. Mice treated with anti-IL10R1 antibody (5,606±1,991 mm^3^) showed a significant, fivefold reduction in CNV volume compared with those receiving the isotype control (26,331±6,192 mm^3^, *P*<0.0075, Student's unpaired *t*-test; Fig. 2a). These findings demonstrate the importance of IL10R-mediated signalling in regulating CNV, and highlight the potential relevance of increased IL10 in macrophages in old mice. Because surface receptor expression is critical in mediating the magnitude of cytokine signalling, we analysed the surface expression of IL10R1 in young and old macrophages by flow cytometry. Our data demonstrated that there was no significant difference in IL10R1 expression between young and old macrophages (Fig. 2b). These findings indicated that the enhanced STAT3 activity observed in old macrophages was not due to increased surface receptor expression, and further corroborated our previous findings implicating the importance of IL10 intracellular signalling in the ageing eye during CNV^12^.

As IL10R1 binds to IL10R2 to facilitate STAT3-mediated signalling, and because old macrophages demonstrated prolonged STAT3 activity after IL10 stimulation, we next investigated the role of IL10R-mediated STAT3 signalling utilizing *IL10r2*^*−/−*^ mice^29^. First, we confirmed that the macrophages from the *IL10r2*^*−/−*^ mouse lacked the ability to respond to IL10 cytokine, as demonstrated by the absence of Y705-STAT3 activation after IL10 treatment (Fig. 2c). Next, we assessed the ability of wild-type (WT) or *IL10r2*^*−/−*^ macrophages to inhibit vascular proliferation *in vitro* using a well-characterized assay of human microvascular endothelial cell (HMVEC) proliferation. We found that *IL10r2* deficiency enhanced the ability of macrophages to inhibit endothelial cell proliferation (63.63% compared with 34.19% relative to HMVEC alone, *P*<0.0001, Student's unpaired *t*-test; Fig. 2d). To establish a link between the phenotype observed in the HMVEC assay and the macrophage polarization of *IL10r2*^*−/−*^ macrophages, we performed qPCR to determine the expression level of M2 polarization markers within these cells. The *IL10r2*^*−/−*^ macrophage expressed significantly lower levels of M2 markers, such as *Arg1* (Fig. 2e) and *Cd163* (Fig. 2f), while expressing higher levels of M1 markers such as *Il6* (Fig. 2g) and *Tnf* (Fig. 2h). Given that IL10 is an M2-promoting cytokine that targets *Cd163* and *Arg1* genes through STAT3 signalling, it is conceivable that the *IL10r2*^*−/−*^ macrophages are less M2-like due to abrogation of IL10/STAT3 signalling^30^,^31^,^32^. Furthermore, *IL10r2*^*−/−*^ macrophages were resistant to M2 polarization because these macrophages failed to increase *Cd163* or *Arg1* expression after IL10 treatment (Supplementary Fig. 2). Taken together, these data demonstrate the importance of IL10R2-mediated signalling in determining macrophage alternative (M2) activation and downstream effects on their ability to regulate vascular proliferation.

### Macrophages deficient in STAT3 inhibit angiogenesis

**Figure 3:**
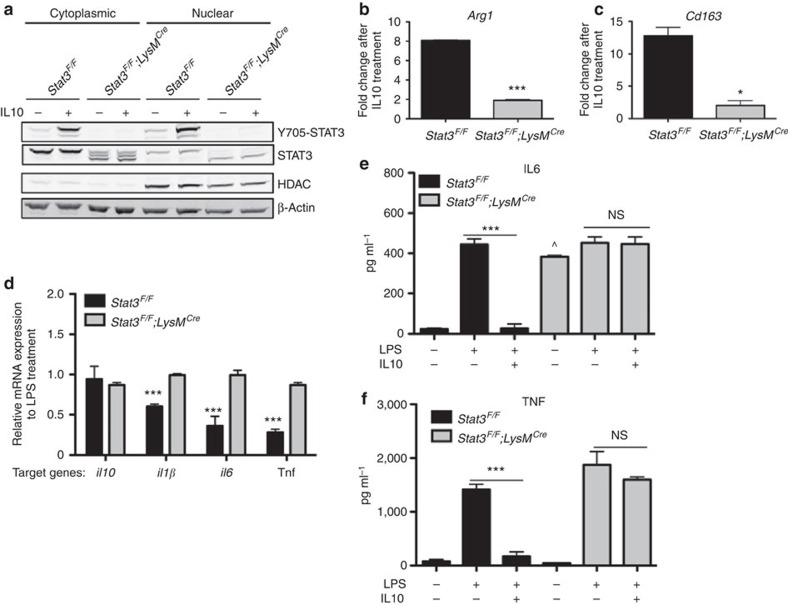
*Stat3*^*F/F*^*;LysM*^*Cre*^ macrophages are resistant to M2 polarization and express persistent pro-inflammatory cytokines. (**a**) Peritoneal macrophages from control (*Stat3*^*F/F*^) and knockout (*Stat3*^*F/F*^*;LysM*^*Cre*^) mice were treated with IL10. Immunoblots for Y705-STAT3 (79/86 kDa) and total STAT3 (79/86 kDa) expression in cytoplasmic and nuclear lysate are shown. β-Actin (42 kDa) and HDAC (60 kDa) were used as loading controls for cytoplasmic and nuclear protein, respectively. Representative data from three independent experiments are shown. (**b**) *Arg1* and (**c**) *Cd163* mRNA fold change in *Stat3*^*F/F*^ or *Stat3*^*F/F*^*;LysM*^*Cre*^ macrophages after 18 h of IL10 treatment in culture was measured by real-time qPCR. Values are expressed in fold change relative to untreated macrophage as mean+s.e.m. At least two biological replicates were analysed for three independent experiments. (Student's unpaired *t*-test; ***P<0.001, *P<0.05 compared with *Stat3*^*F/F*^ mice). (**d**) Peritoneal macrophages were treated with LPS or LPS and IL10 co-treatment for 18 h. Real-time qPCR was performed to measure relative suppression of inflammatory cytokines, *Il1β*, *Il6* and *TNF*. (*N*>3 pooled macrophage samples in independent experiments are shown, Student's unpaired *t*-test, ***P<0.001 compared with LPS alone). ELISA for (**e**) IL6 and (**f**) TNF was performed to determine the cytokine concentration in the media after LPS or LPS and IL10 co-treatment. *N*=3 each; Student's unpaired *t*-test, ***P<0.001 compared with LPS alone, NS (not significant) *P*>0.05, compared with LPS alone, ^*P*<0.001 compared with *Stat3*^*F/F*^.

Both anti-IL10R1 antibody-mediated rescue and experiment using *IL10r2*^*−/−*^ mouse are global approaches to examine polarization and effector function but lack cell type specificity. Thus, it is important to delineate whether IL10 signalling specifically in macrophages plays a role in regulating CNV. To do this, we generated a conditional knockout mouse lacking functional STAT3 in the myeloid cell lineage including monocytes, mature macrophage and granulocytes (*Stat3*^*F/F*^*;LysM*^*Cre*^)^26^,^33^,^34^. We first verified that IL10-mediated STAT3 signalling is abrogated in the knockout macrophage by performing a series of biochemical characterizations in peritoneal macrophages. Using both western blotting and immunohistochemistry approaches, we demonstrated that *Stat3*^*F/F*^*;LysM*^*Cre*^ macrophages are unable to respond to IL10 via STAT3 signalling as indicated by the lack of STAT3 phosphorylation as well as its nuclear translocation (Fig. 3a and Supplementary Fig. 3). To determine the precise role of STAT3 activity in the sequential events leading to macrophage polarization, macrophages were treated with LPS or IL10, and changes in their activation states were analysed by quantitative gene expression and enzyme-linked immunosorbent assay (ELISA). STAT3 deficiency in macrophages significantly inhibited the induction of M2 markers such as *Arg1* and *Cd163* on IL10 stimulation, confirming the key role of STAT3 as a mediator of IL10 signalling and M2 polarization (Fig. 3b,c). Conversely, LPS treatment induced pro-inflammatory cytokine expression, including *IL1β*, *Il6* and *Tnf*. Interestingly, co-treatment with IL10 effectively reduced the mRNA (Fig. 3d) and protein (Fig. 3e,f) levels of these cytokines in control macrophages, but not in the *Stat3*^*F/F*^*;LysM*^*Cre*^ macrophages, suggesting a deficiency in physiological feedback signalling that is critical for homeostatic regulation of macrophage function. These data suggest that genetic ablation of STAT3 signalling activity in macrophages leads to sustained expression of pro-inflammatory cytokines (Supplementary Fig. 4) that is unresponsive to the anti-inflammatory effects of IL10.

**Figure 4:**
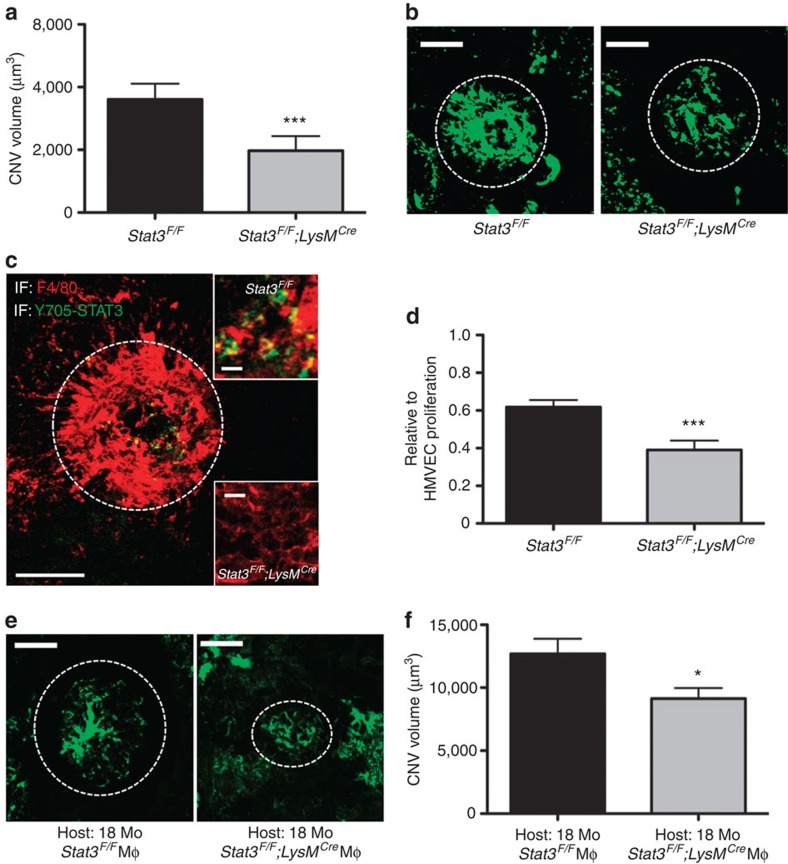
*Stat3*^*F/F*^*;LysM*^*Cre*^*mice* are protected against neovascular proliferation. (**a**) Laser-induced CNV was performed on *Stat3*^*F/F*^*;LysM*^*Cre*^ and *Stat3*^*F/F*^ mice. Mice were perfused with fluorescein isothiocyanate–dextran to visualize CNV complex and quantified. Values are expressed as mean+s.e.m. *N*>16 eyes for each genotype. Student's unpaired *t*-test. ***P<0.00075 compared with *Stat3*^*F/F*^ mice. (**b**) Representative CNV pseudo-volumetric images for *Stat3*^*F/F*^ and *Stat3*^*F/F*^*;LysM*^*Cre*^ are shown. Areas of CNV quantified are indicated in white dotted circles. Scale bars, 20 μm. (**c**) A representative immunohistochemical image of F4/80 (red) and activated Y705-STAT3 (45) in CNV lesion. Infiltrating F4/80^+^ macrophages also express active STAT3 signalling in control but not in *Stat3*^*F/F*^*;LysM*^*Cre*^. White dotted circle indicates the proportional size of CNV complex. Scale bars on the main picture and insets are 100 and 10 μm, respectively. (**d**) HMVEC proliferation assay demonstrates *Stat3*^*F/F*^*;LysM*^*Cre*^-enhanced inhibition of HMVEC proliferation *in vitro* compared with control macrophages. Values are reported as mean+s.e.m. relative to lone HMVEC culture. *N*=20 co-cultures for each genotype. Student's unpaired *t*-test. ***P<0.0005. (**e**) Representative CNV pseudo-volumetric images for 18-month (Mo)-old mice receiving *Stat3*^*F/F*^ or *Stat3*^*F/F*^*;LysM*^*Cre*^ macrophage followed by laser-induced CNV are shown. Areas of CNV quantified are indicated in white dotted circles. Scale bars, 20 μm. (**f**) Quantification of CNV volume in macrophage adoptive transfer experiment. Values are expressed as mean+s.e.m. *N*>11 eyes for each genotype. Student's unpaired *t*-test. *P<0.0199 compared with mice receiving control s*tat3*^*F/F*^ macrophage.

Previous studies have shown that an IL10-rich environment in the eye is amenable to alternative (M2) macrophage activation and development of CNV^14^,^16^. Thus, we explored the implications of this M2 resistant and M1 prone character of *Stat3*^*F/F*^*;LysM*^*Cre*^ macrophage *in vivo*. When *Stat3*^*F/F*^*;LysM*^*Cre*^ mice were examined in the injury-induced CNV model, there was a marked decrease in CNV volume compared with littermate controls (7,598±1,061 versus 4,138±996 mm^3^, *P*<0.0075, Student's unpaired *t*-test; Fig. 4a,b). To further corroborate the role of STAT3 signalling within the CNV complexes in the eye, we performed immunohistochemistry to demonstrate that the reduction in CNV was associated with an absence of STAT3 activation in the infiltrating F4/80^+^ macrophages (Fig. 4c). To demonstrate the cellular and functional role of STAT3 signalling in macrophages, we performed an HMVEC proliferation assay. *Stat3*^*F/F*^*;LysM*^*Cre*^ macrophages demonstrated an enhanced ability to inhibit HVMEC proliferation compared with littermate controls (61.81 versus 39.00% relative to HMVEC alone, *P*<0.001; Fig. 4d). Interestingly, the level of inhibition was similar to that obtained when using macrophages depleted for either the *Il10r1* or *Il10r2* genes, which further highlights the significant and critical coordination between IL10 and STAT3 signalling in the vascular proliferation (Fig. 2d and ref. 14).

Finally, to further demonstrate the pro-angiogenic role of STAT3 signalling in macrophage, we performed an intravitreal injection of control *Stat3*^*F/F*^ or *Stat3*^*F/F*^*;LysM*^*Cre*^ macrophages into 18 months old mice after CNV induction. Quantification of choroidal neovascular complexes revealed that mice receiving *Stat3*^*F/F*^*;LysM*^*Cre*^ macrophages developed significantly less CNV compared with the mice that received control macrophages (Fig. 4e,f). These results conclusively demonstrate that STAT3 activation is crucial for macrophage regulation of pathological vascular proliferation and CNV.

As shown above, STAT3 signalling activity is highly associated with the alternative (M2) polarization of macrophages, which confirms previous observations regarding angiogenesis in tumour-associated macrophages^35^,^36^,^37^. NF-κB and STAT3 signalling pathways are the two main molecular cascades known to govern M2 polarization in tumour-associated macrophages^9^,^38^. In the eye, there are a number of reports suggesting a role for NF-κB activation in the regulation of CNV^39^,^40^. However, our data indicated that the NF-κB pathway in macrophages did not play a role in CNV, as we did not observe a significant difference in NF-κB signalling between young and old macrophages (Fig. 1d). In addition, macrophage-specific knockouts of IKKβ (inhibitor of kappa light polypeptide gene enhancer in B-cells, kinase beta) showed no effect on CNV *in vivo* or vascular proliferation *in vitro* (Supplementary Fig. 5).

### 5,15-DPP is a potent inhibitor of STAT3-driven angiogenesis

**Figure 5:**
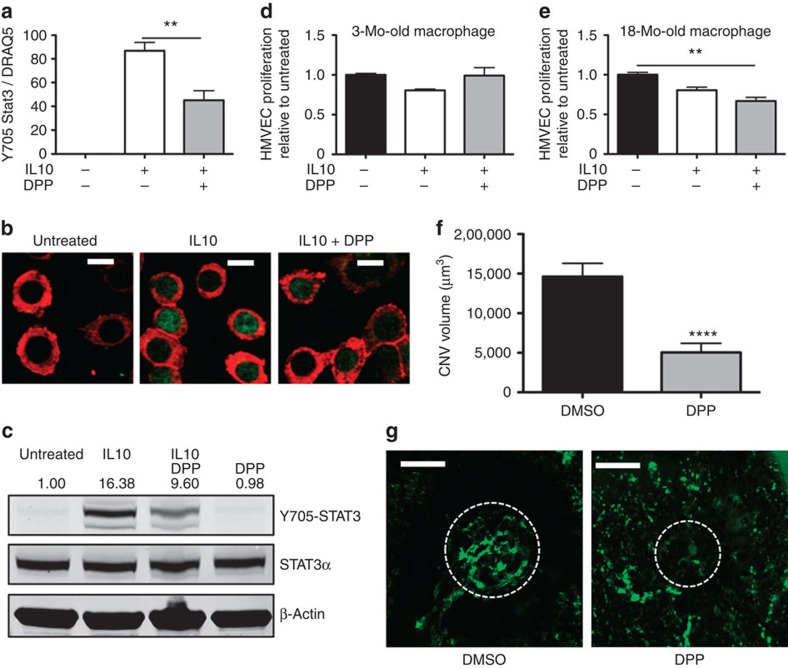
5,15-DPP is an effective STAT3 inhibitor and blocks IL10-mediated signalling in macrophages leading to altered regulation of CNV. (**a**) 264.7 RAW cells were treated with IL10 or IL10 with 5 mM 5,15-DPP for 1 h. Immunohistochemical analysis was used to visualize and quantify nuclear Y705-STAT3 expression and normalized to total DRAQ5 nuclear-positive cells. Values are expressed as mean+s.e.m. from a minimum of five independent cultures. One-way analysis of variance with a Tukey's multiple comparison test. **P<0.01 compared with IL10 treated. (**b**) Representative immunohistological images of RAW cells stained for F4/80 (red) and Y705-STAT3 at baseline, after IL10 treatment and IL10 and DPP co-treatment. Scale bars, 20 μm. (**c**) Representative immunoblots of Y705-STAT3 (79/86 kDa), STAT3 (86 kDa) and β-actin (42 kDa) in whole-cell lysate from untreated, IL10 treated, IL10 and DPP co-treated, and DPP-treated macrophages whole-cell lysate. (**d**) Young or (**e**) old peritoneal macrophages were untreated, pretreated with IL10 or co-treated with IL10 and 5,15-DPP before HMVEC proliferation assay. Values are expressed as mean+s.e.m. relative to untreated cultures. *N*=4. One-way analysis of variance with a Tukey Kramer post test. **P<0.01 compared with untreated group. (**f**) Eighteen-month (Mo)-old female mice were either treated with daily DMSO or 5,15-DPP (15 mg kg^−1^) injections for 5 days before and 6 days after CNV injury. Mice were perfused with fluorescein isothiocyanate–dextran to visualize CNV complex and to quantify CNV volume. Values are expressed as mean+s.e.m. *N*=9 eyes for each treatment group. Student's unpaired *t*-test. ****P<0.0001 compared with DMSO-treated mice. (**g**) Representative CNV pseudo-volumetric image of mouse treated with DMSO or DPP. Areas of CNV quantified are indicated by white dotted circles. Scale bars, 20 μm.

Having established the foundation that STAT3 is a key regulator of macrophage activity and function, we sought to reverse the enhanced CNV phenotype and ameliorate disease burden in old mice by targeting STAT3 signalling. Pharmacological STAT3 inhibitors have been popular targets for drug development owing to their potential applications in cancer and liver diseases^41^,^42^. Several studies have demonstrated efficacy of targeting infiltrating macrophages within the CNV lesions^13^,^14^,^43^. To test the therapeutic benefit of targeting STAT3 in CNV, we used 5,15-diphenyl-porphine (DPP) because of its high specificity to STAT3 over other STAT proteins^44^. Moreover, the drug has been previously assessed in an animal model for partial hepatectomy, wherein mice showed excellent drug tolerance to treatment^45^. We first determined the effect of the 5,15-DPP-mediated targeting of STAT3 signalling using a murine macrophage cell line (RAW264.7). Co-treatment of 5,15-DPP with IL10 resulted in a significant reduction (85 versus 45%, *P*<0.002, Student's unpaired *t*-test) in nuclear Y705-STAT3 expression observed using both immunohistochemistry and western blotting (Fig. 5a–c).

To validate the functional efficacy of the 5,15-DPP-mediated inhibition of STAT3, both young and old macrophages were treated with IL10 alone or in combination with 5,15-DPP. Cells were then washed before co-culture with HMVEC cells. 5,15-DPP successfully restored the ability of old macrophage to inhibit vascular proliferation, but this beneficial effect was not observed in young macrophages (Fig. 5d,e). The differential drug efficacy in young macrophages compared with old could be explained by their STAT3 activation state. Indeed, in young macrophages, the low response to IL10 treatment in terms of subsequent activation of STAT3 (Fig. 1d) limited the effectiveness of 5,15-DPP and its impact on macrophage function. In addition, the active IL10/STAT3 signalling pathway state and an M2-like phenotype as seen in old macrophage likely played a role in the efficacy of 5,15-DPP. These data suggest that 5,15-DPP-mediated inhibition of STAT3 signalling in old macrophages is therapeutic and restores the capacity of these cells to inhibit vascular proliferation, a crucial process involved in development of CNV.

To determine whether the anti-angiogenic and therapeutic effects of 5,15-DPP could be demonstrated *in vivo*, we investigated its effect on CNV. Old mice were pretreated with either vehicle (dimethylsulphoxide, DMSO) or 5,15-DPP via intraperitoneal (i.p.) injection for 5 days before laser injury, and the mice continued to receive daily injections until analysis for CNV volume on day 7. The drug-treated animals demonstrated a significant reduction of CNV by almost threefold versus DMSO-treated controls (13,873±1,601 versus 5,151±955 mm^3^, *P*<0.0001, Student's unpaired *t*-test; Fig. 5f,g). This therapeutic benefit can be at least partially attributed to the reduced STAT3 signalling in infiltrating macrophages because western blot analysis of peritoneal macrophages isolated from mice treated with 5,15-DPP showed lower STAT3 signalling activity compared with DMSO controls (Supplementary Fig. 6). Taken together, these experiments have established that the ability to switch phenotypic state of old macrophages is a valuable tool to reprogramme them into anti-angiogenic cells with therapeutic potential.

### SOCS3 is reduced in old macrophages

**Figure 6:**
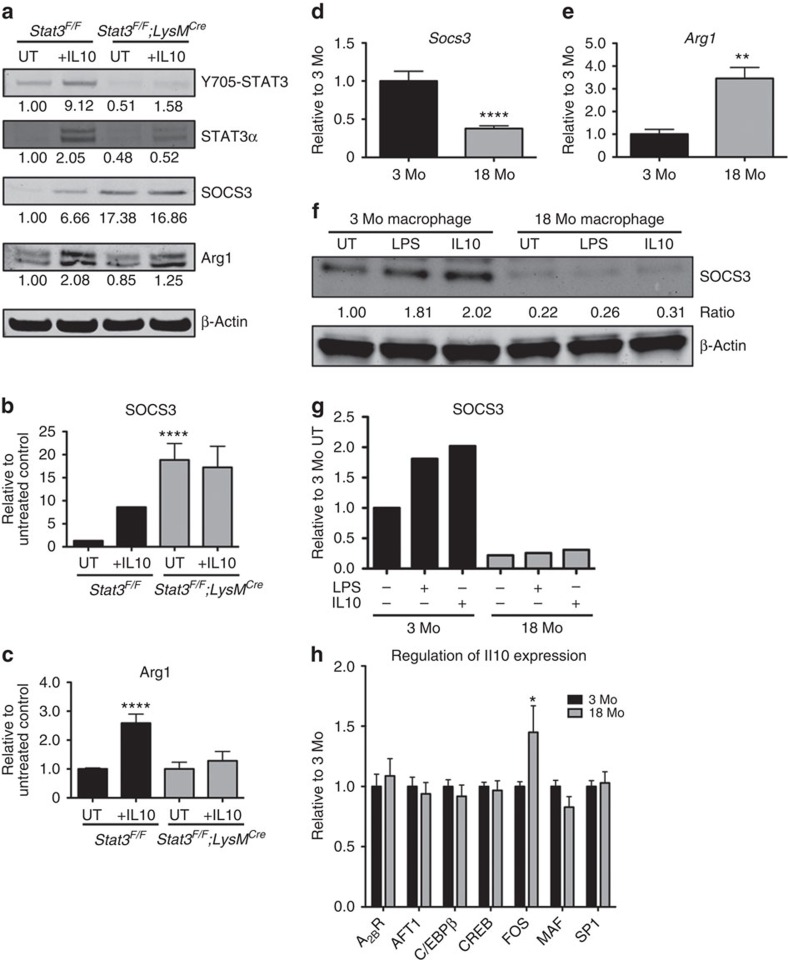
SOCS3 is a key regulator of the CNV phenotype. (**a**) *Stat3*^*F/F*^ and *Stat3*^*F/F*^*;LysM*^*Cre*^ macrophages were treated with IL10 for 18 h. Representative immunoblots for Y705-STAT3 (79/86 kDa), STAT3α (86 kDa), SOCS3 (27 kDa) and Arg1 (40 kDa) are shown. Relative (**b**) SOCS3 (26 kDa) and (**c**) Arg1 (40 kDa) expression to β-actin (42 kDa) was normalized to untreated (UT) Stat3^F/F^ samples. Mean values are indicated underneath each blot. *N*>2 biological replicate per group. Real-time qPCR for (**d**) *Socs3* and (**e**) *Arg1* expression in young and old macrophages. Values are expressed as mean+s.e.m. *N*>5 biological replicates, ****P<0.0001, **P<0.01, Student's unpaired *t*-test, compared with 3-month (Mo)-old macrophage. Young and old peritoneal macrophages were treated with LPS or IL10 for 18 h. (**f**) Representative immunoblots for SOCS3 and β-actin expression from whole-cell lysate. (**g**) Relative SOCS3 expression to β-actin was normalized to 3 Mo macrophage that was UT. *N*>2 biological replicate per group. (**h**) Quantitative mRNA analysis of adenosine 2B receptor (A_2B_R), activating transcription factor 1 (ATF1), CCAAT/enhancer binding protein-β (C/EBPβ), cAMP response element-binding protein (CREB), FOS, MAF and specific protein 1 (SP1) in peritoneal macrophages isolated from young or old macrophages.

Suppressor of cytokine signalling 3 (SOCS3) is a well-known target of STAT3-driven signalling after IL10 stimulation and acts as a feedback regulator of IL10 production^46^. It was recently reported that SOCS3 acts as an endogenous anti-angiogenic factor in endothelial cells during retinopathy of prematurity^47^. Based on these findings, we sought to determine whether the SOCS3 regulatory pathway was involved in the effects of STAT3 signalling on macrophage polarization and CNV. First, we evaluated SOCS3 expression in *Stat3*^*F/F*^*;LysM*^*Cre*^ and control macrophages after IL10 treatment. As shown in Fig. 6a, IL10 treatment in control macrophages induced both SOCS3 and Arg1 expression by over six- and twofold, respectively, suggesting that under normal conditions in young macrophages, SOCS3 may be co-activated by IL10 treatment along with other M2 markers such as Arg1. Interestingly, while *Stat3*^*F/F*^*;LysM*^*Cre*^ macrophages did not respond to IL10 treatment, as evidenced by a lack of changes in SOCS3 or Arg1 expression, the baseline expression of SOCS3 was significantly higher than littermate controls (Fig. 6a–c).

The differential SOCS3 expression in STAT3 knockout macrophages led us to investigate whether baseline expression of SOCS3 determined the responsiveness to IL10 cytokine and the strong M2-like phenotype found in old macrophages. Analysis of *Socs3* gene expression in young and old macrophages revealed that old macrophages expressed significantly lower SOCS3 and higher levels of M2 marker genes, such as *Arg1*, than young macrophages, as measured by real-time qPCR (Fig. 6d,e), demonstrating an inverse relationship between classical M2 markers and SOCS3 expression in aged macrophages. As SOCS3 is also known to be a target gene regulated by STAT3 signalling after LPS or IL10 treatment, we investigated how age affected responsiveness to LPS and IL10 with respect to SOCS3 induction^46^. When young macrophages were treated with either LPS or IL10, SOCS3 protein levels were significantly elevated. On the other hand, induction of SOCS3 in old macrophages after LPS or IL10 treatment was minimal (Fig. 6f,g). These data suggest that old macrophages exhibited not only a lower baseline expression level of SOCS3 but also defective regulation of SOCS3 expression on IL10 treatment resulting in enhanced STAT3 activity. These results provide new evidence implicating dysregulated SOCS3 expression and activity in old macrophages as a possible mechanism contributing to sustained STAT3 activation and ultimately leading to M2 polarization. To further investigate the age-dependent increase of IL10 production in macrophages, we examined the levels of several molecular regulators of IL10 expression in immune cells^48^,^49^. In senescent macrophages, the expression of the transcription factor FBJ osteosarcoma oncogene (FOS) is significantly higher as compared with young macrophages (Fig. 6h). The upregulation of FOS may explain the anti-inflammatory profile of aged macrophages associated with increased IL10 production.

### Activation of macrophage STAT3 signalling in AMD patients

**Figure 7:**
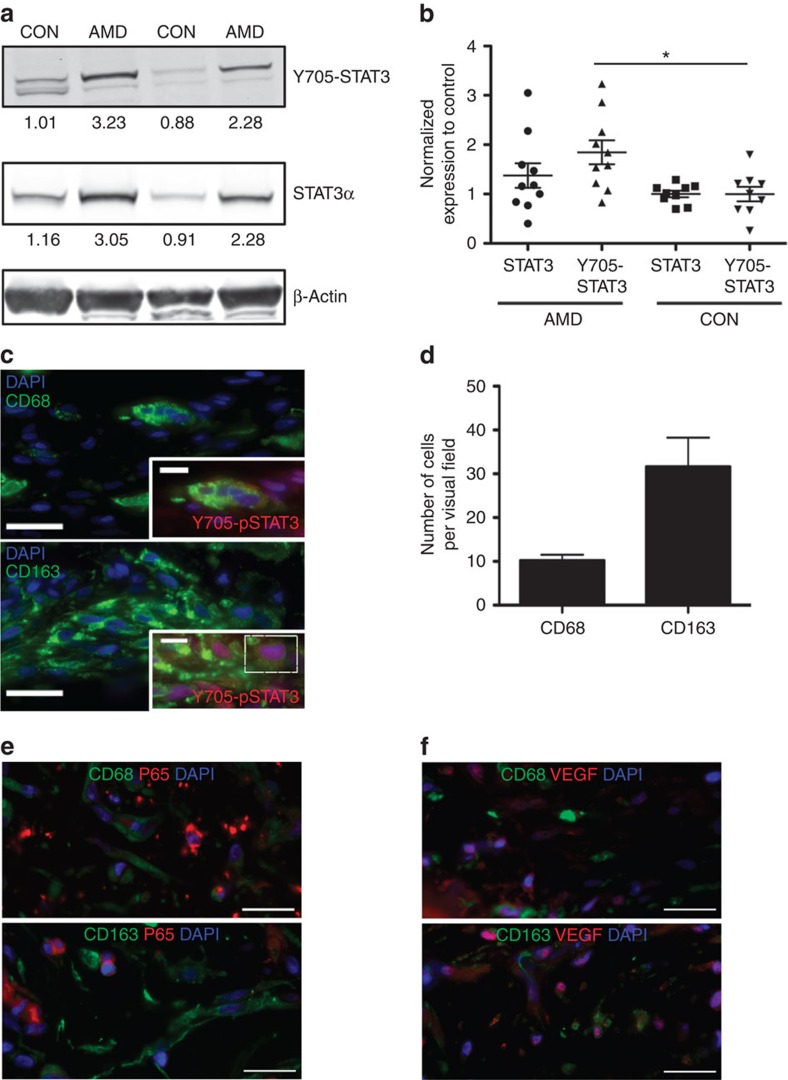
Higher STAT3 activation is observed in PBMCs and in CD163-positive eye macrophages of patients with wet AMD. (**a**) Human PBMCs were isolated from wet AMD or age-matched non-AMD (CON) donors. Whole-cell lysates were then subjected to western blotting analysis. Two representative blots from each group are shown. Relative value of protein expression to β-actin was normalized to CON as shown underneath each blot and (**b**) quantification of immunoblots is shown. (*n*>5 donors samples, *P<0.05, Student's unpaired *t*-test, compared with age-matched non-AMD donors). (**c**) Choroidal neovascular membrane of human donor was subjected to immunohistochemical analysis for the presence of CD68+ (green, top) and CD163+ (green, bottom). Insets show higher magnification of cells co-stained with polarization markers and nuclear Y705-STAT3 (red) in CD163+ cell. DAPI (blue) was used to counter stain the nuclei. Scale bars are 20 and 5 μm for lower and higher magnification, respectively. (**d**) Quantification of CD68 and CD163 in human choroidal neovascular membrane. Graph shows the mean+s.e.m. of cells identified in a given visual field, *N*=6 independent sections. (**e**) Choroidal neovascular membranes were stained with antibodies against P65 and macrophage markers CD68 (upper panel) or CD163 (lower panel). (**f**) VEGF immunostaining of choroidal neovascular membranes and dual-labeling with anti-CD68 or CD163. Nuclei were stained with DAPI. Scale bars, 20 μm.

The above experiments demonstrate that during ageing, excessive IL10 expression and STAT3 activation are the determinants of M2 macrophage polarization and the pro-angiogenic and disease-promoting phenotype observed in mice. In the next set of experiments, we investigated whether these cellular and molecular phenotype findings are relevant to the monocytes/macrophages of patients diagnosed with neovascular AMD. To do this, we examined STAT3 activation in human peripheral blood mononuclear cells (PBMCs) isolated from wet AMD (age range 67–87 years, *n*=10) and non-AMD age-matched donors (58–77 years, *n*=9). Western blot analysis displayed a remarkably higher expression of phospho-STAT3 proteins in AMD patients compared with controls (Fig. 7a,b), confirming the findings in mice and suggesting a link between STAT3 activation in macrophage and neovascular AMD.

Because there is no clear evidence that PBMCs in humans necessarily reflect the disease pathophysiology in CNV in eyes of patients with AMD, we further characterize the involvement of STAT3 activation *in situ* using an immunohistochemical approach. Choroidal neovascular membranes surgically removed from AMD patients as part of their usual therapy were stained to investigate the infiltrating macrophage subpopulation. CD68 and CD163 are widely accepted markers used to identify human M1 and M2 macrophage populations, respectively, in numerous age-related diseases^50^,^51^,^52^. Using these surface marker proteins as reference, we noted that the majority of cells that were CD163-positive expressed nuclear phospho-STAT3 protein while CD68-positive macrophages did not express prominent phospho-STAT3 expression (Fig. 7c). In addition, the number of M2 macrophages was far greater than M1 macrophages in the neovascular membranes (Fig. 7d). These immunohistochemical data are a strong indication of a potential relationship between STAT3 signalling and the M2 macrophage phenotype found in CNV from eyes of patients with AMD. Although CD68+ and CD163+ macrophages were detected in neovascular membranes, there was no activation and nuclear translocation of P65-NF-κB (Fig. 7e). These findings further confirm that in macrophages, NF-κB signalling pathway is not critical for the regulation of CNV (Supplementary Fig. 5). Given the central role of VEGF in modulating pathological vascularization^53^, we analysed its cell-specific production in CNV membranes. Our current data indicate that VEGF is secreted in the neovascular complex and its production is not restricted to a macrophage subpopulation (Fig. 7f). VEGF staining was observed in CD68+ macrophages and CD163+ macrophages.

## Discussion

**Figure 8:**
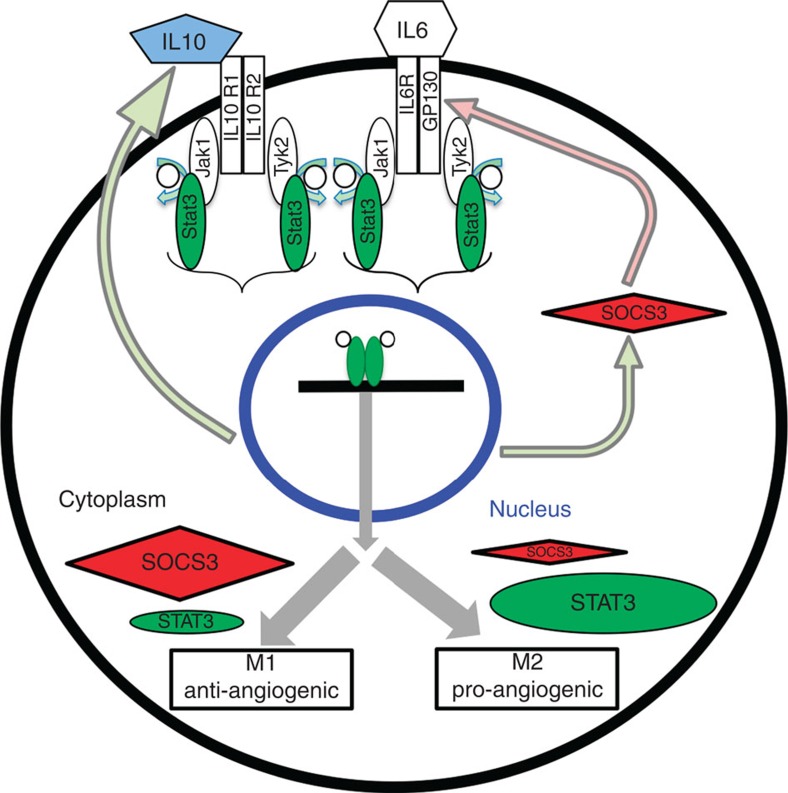
Mechanism of STAT3-mediated regulation of macrophage polarization and their ability to regulate angiogenesis. Proposed role of baseline SOCS3 (red) expression and IL10 (blue)-mediated STAT3 (green) signalling in ageing macrophages. Low baseline SOCS3 expression in old macrophages leads to increased susceptibility to IL10-mediated STAT3 signalling that polarizes macrophages towards M2 alternative activation. This change in polarization promotes abnormal angiogenesis.

The present study demonstrates that STAT3 signalling is an important determinant of alternative M2 activation in senescent macrophages and promotes angiogenesis in blinding eye diseases (Fig. 8). First, old macrophages are exposed to an IL10-rich micro-environment in the eye and are more susceptible to IL10 cytokine stimulation, as evidenced by prolonged STAT3 activation. Second, loss-of-function approaches targeting the IL10/STAT3 pathway utilizing genetic and antibody-mediated modalities resulted in a decrease in angiogenesis *in vivo* and *in vitro*. Third, macrophages with either dysfunctional *Il10r2* or *Stat3* genes displayed lower expression of M2 markers such as *Arg1* and *Cd163*, and these cells were resistant to alternative M2 activation after IL10 stimulation. Fourth, M2 polarization and the disease-promoting pro-angiogenic environment in the eyes of old mice was pharmacologically rescued by 5,15-DPP, a potent inhibitor of STAT3. Fifth, we discovered that baseline expression of SOCS3 is a critical component that predicts macrophage polarization within an IL10-rich environment. Last and perhaps most relevant, human choroidal neovascular membranes obtained from eyes of AMD patients were not only rich with CD163^+^ M2 macrophages but these macrophages also expressed nuclear phospho-STAT3 confirming activation. Taken together, we have elucidated the role of STAT3 signalling in regulating M2 alternative activation in senescent macrophages and their function in impaired regulation of angiogenesis in blinding eye diseases such as AMD.

It has been consistently demonstrated that senescent macrophages express high levels of M2 markers such as IL10, Arg1 and CD163 accompanied with reduced levels of M1 markers such as IL1β, tumour-necrosis factor (TNF)-α, chemokine (C-C motif) ligand 2 (CCL2), matrix metallopeptidase 9 (MMP9) and IL6 compared with young at baseline^14^,^26^. Our data indicate that the balance between SOCS3 and STAT3 signalling is compromised in senescent macrophages and which might explain why old mice are more susceptible to CNV. In previous studies, it has been demonstrated that pro-inflammatory cytokine IL6 and anti-inflammatory cytokine IL10 share the same STAT3 signalling pathway, which induces SOCS3 expression. SOCS3 then targets GP130, a receptor for IL6, but not IL10R, and this difference in receptor targeting by SOCS3 results in shorter IL6-driven STAT3 activity, while IL10-driven STAT3 activation is prolonged^54^. We speculate that decreased SOCS3 expression in the senescent macrophages favours more prolonged IL10/STAT3 signalling. The importance of this kinetic difference has been reported in previous thermodynamic and biological experiments^54^,^55^. Under pathological conditions, reduced expression of SOCS3 has been associated with various human cancer cells where STAT3 is constitutively activated^56^,^57^,^58^. Therefore, further investigation is required to understand the regulation of SOCS3 in pathological states.

Identification of markers for alternatively (M2) activated macrophages is an important aspect of defining the precise macrophage cell type so as to investigate disease pathogenesis accurately. In our study, the selective usage of CD163 and Arg1 as markers for M2 alternatively activated macrophages with STAT3 signalling helped us hone in on the direct relationship between CD163 and STAT3 activity. The CD68 transmembrane protein marker has been used to identify macrophages in human pathology. It is particularly interesting that inactivation of STAT3 polarize macrophages to a pro-inflammatory phenotype associated with a higher expression of M1 markers. An earlier study reported that CD68-positive macrophages isolated from human CNV co-express TNF and IL1-β, but not VEGF^52^. The negative regulation of STAT3 signalling on TNF expression is a well-characterized phenomenon: thus, the CD68 macrophage characteristics observed in our study and previously reported by Oh *et al.* support our current model of alternative (M2) activation of the senescent macrophage in human disease^12^,^51^. More interestingly, infiltration of CD68-positive macrophage has been associated with AMD patients that received intraocular anti-VEGF therapy^59^. It is tantalizing to speculate that the therapeutic benefits of the anti-VEGF agents may be partly attributable to an anti-angiogenic effect of infiltrating CD68^+^ M1 macrophages in the eye.

Interestingly, adoptive transfer of STAT3-deficient macrophages in the eye of old mice resulted in significant reduction of pathological angiogenesis. The ability of these macrophages to inhibit CNV in a pro-angiogenic micro milieu demonstrated how macrophage polarization induced by STAT3 signalling regulates vascular proliferation in a pathological context.

In summary, our studies show that age-associated changes in IL10-driven STAT3 signalling promote alternative (M2) polarization in old macrophages. Both retinal micro-environmental changes and autonomous changes in macrophage response to IL10 signalling contribute to a pro-angiogenic M2 phenotype. In our models of angiogenesis, sustained STAT3 signalling in senescent macrophages channels M2 polarization and promotes neovascularization. These finding are reproducible both systemically in PBMCs and eye macrophages obtained from human patients with blinding eye disease AMD characterized by aberrant neovascularization. Of significant interest is the finding that impaired regulatory function and polarization of old macrophages is therapeutically modifiable. Together, these data suggest that M2 macrophage-specific targeting of STAT3 signalling pathway may lead to novel therapies to treat CNV in AMD and potentially other diseases such as atherosclerosis and cancers where abnormal proliferative angiogenesis is pathogenic.

## Methods

### Animals

All animal experiments were approved by the Animal Studies Committee of Washington University in St Louis School of Medicine, and performed under all Animal Care and Use guidelines. Young female C57BL/6J mice (age <3 months) and old female C57BL/6J mice (age >18 months) were purchased from The Jackson Laboratory (Bar Harbor, ME) and the National Institute of Aging, respectively. *Il10r2*^*−/−*^ mice (C57BL/6J) were generously provided by Dr Paul Allen from Washington University in St Louis. The macrophage-specific *Stat3*^*F/F*^*;LysM*^*Cre*^ (C57BL/6J) line was generated by breeding *Stat3*^*F/F*^ mice, provided by Dr Shizuo Akira (Osaka University, Japan^60^), with *LysM*^*Cre*^ mice (The Jackson Laboratory). The *Ikkβ*^*F/F*^ (C57BL/6J) mouse line was provided by Dr Michael Karin of University of California San Diego^61^. All mice bred and used for these experiments (males and females) were screened for *rd1* and *rd8* mutations using published protocols to assure that only mice with a WT background were used for the experiments^62^,^63^.

### Cell culture and human PBMCs

The mouse monocyte/macrophage RAW264.7 cell line was purchased from American Type Culture Collection (ATCC) and cultured in Dulbecco's minimum essential medium (Life Technologies, Carlsbad, CA) with 10% fetal calf serum (Atlanta Biologicals, Flowery Branch, GA), 1% penicillin and streptomycin (Life Technologies), 1% non-essential amino acids (Life Technologies), and 1% sodium pyruvate (Life Technologies) and GlutaMax (Life Technologies). Dermal HMVECs were purchased from Lonza (Walkersville, MD) and cultured in EGM2V media. Peritoneal macrophages were isolated 5 days after i.p. injection of 4% thioglycollate (Sigma-Aldrich, St Louis, MO). Isolated macrophages were allowed to recover in RPMI-1640 media (Life Technologies) with 10% FBS, and 1% penicillin and streptomycin overnight. Cells were then scraped and replated according to the protocol described below. Human PBMCs were isolated from donors with neovascular AMD or age-matched controls. This study was approved by the Human Research Protection Office of Washington University School of Medicine in St Louis, and informed consent was obtained from all blood donors. PBMCs were first collected in a Cell Preparation Tube with sodium citrate Vacutainer (Becton, Dickinson and Company, Franklin Lakes, NJ) PBMCs were isolated and washed according to the manufacturer's protocol and stored in −80 °C until experimental use.

### HMVEC proliferation assay

HMVEC proliferation assay was performed according to previously described methods^26^,^64^. Briefly, HMVEC (3 × 10^3^ cells) were cultured in 96-well round-bottomed plates and allowed to adhere to the plate overnight. Cells were then co-cultured with peritoneal macrophage (1:25 ratio) and media containing 1 μCi ml^−1^ [^3^H] thymidine (PerkinElmer, Shelton, CT). For the STAT3 inhibition experiment, macrophages were cultured in media containing 5 μM 5,15-DPP (Sigma-Aldrich) overnight, washed with PBS, and viable cells were counted before being co-cultured with HMVECs. After 18 h, cells were harvested onto glass fibre filters (Packard) and incorporated [^3^H] thymidine was counted using TopCount (PerkinElmer).

### Real-time PCR and gene expression analysis

Total RNA was prepared from spleen, peritoneal macrophages, retina and choroid using either the RNeasy mini kit or the RNA universal kit (Qiagen, Valencia, CA). Isolated RNA was quantified, and 2.0 μg of RNA was converted to cDNA using the High Capacity cDNA Reverse Transcription kits (Applied Biosystems). PCR amplification of cDNA was performed using Taqman probe-based gene expression assay (Applied Biosystems) as previously described. The following probe sets were used: *ActB*, Mm00607939_s1*; Tnfα*, Mm00443258_m1; *IL6*, Mm00446190_m1; *Il1β*, Mm01336189_m1; *Ptgs2*, Mm00478374_m1; *Ccl2*, Mm00441242_m1; *Mmp9*, Mm00442991_m1; *IL10*, Mm99999062_m1; *Cd163*, Mm00474091_m1; *Nos2*, Mm00440502_m1; *IL4*, Mm00445259_m1; *IL13*, Mm0043434204_m1; Arg1, Mm00475988_m1 and *Socs3*, Mm00545913_s1. Gene expression levels were normalized to a housekeeping gene either *ActB* or *Gapdh* followed by Student's unpaired *t*-test for statistical analysis. For the gene expression analysis after IL10 and LPS treatment fold change in untreated samples were used to further normalize to LPS-treated samples.

### ELISA

Peritoneal macrophages (1 × 10^6^) were seeded onto six-well plates and cultured overnight. Cells were then treated with 100 ng ml^−1^ LPS, 100 ng ml^−1^ LPS plus 100 ng ml^-1^ of recombinant IL10 or left untreated. Media were collected and processed for ELISA according to the manufacturer's protocol (R&D Systems, Minneapolis, MN). Media samples were diluted so the signal fell within the dynamic range of detection established in the standard curve provided. Concentration of cytokines was further normalized to the total protein of cells where culture media was collected. The experiment was repeated three times.

Blood was collected from the mandibular vein of young or old mice and further processed to separate the serum according to the manufacturer's protocol (BD, NJ). Samples were then processed for IL10 ELISA (R&D Systems).

### Immunohistochemistry

Peritoneal macrophages or RAW cells were cultured on chambered glass-bottomed slides (Nalgene Nunc International, Rochester, NY). Cells were then treated with 100 ng ml^−1^ LPS (Sigma-Aldrich), 100 ng ml^−1^ IL10 (Peprotech, Rocky Hill, NJ), a combination of both, or 5 mM 5–15-DPP (Sigma-Aldrich). After treatment, cells were processed for immunostaining according to the manufacturer's protocol (Cell Signaling Technology, Beverly, MA). Briefly, cells were washed with PBS and fixed with 4% paraformaldehyde (Electron Microscopy Sciences, Hatfield, PA) followed by cold 100% methanol (Sigma-Aldrich). Cells were then blocked with 5% goat serum (Sigma-Aldrich) plus 0.3% Triton X-100 (Sigma-Aldrich) for 1 h and incubated with primary antibodies against Y705-Stat3 (1/200; no. 9145, Cell Signaling Technology), NF-κB P65 (1/400; no. 8242, Cell Signaling Technology) and/or F4/80 (1/500; MCA497G, Serotec, Raleigh, NC) overnight at 4 °C. Cells were then washed and incubated with the secondary antibody conjugated with Alexa-488 (1/500) or Alexa-546 (1/500). After the last wash, cells were covered with mounting media containing DAPI (4,6-diamidino-2-phenylindole; Vectashields, Vector Laboratory, Burlingame, CA) or DRAQ5 (no. 4084, Cell Signaling Technology) for nuclear staining and sealed with glass coverslip for confocal analysis with a Zeiss LSM510 microscope (Thornwood, NY). Acquired images were then processed in ImageJ (National Institutes of Health, Bethesda, MD) to create either a single-filter image or a merged image.

For human choroidal neovascular membrane staining, 4 μm paraffin sections were deparaffinized, rehydrated with graded ethanol and subjected to antigen retrieval in citric acid-based unmasking solution (Vector Labs) for 3 min in a decloaking chamber (Biocare Medical, Concord, CA). After cooling at room temperature (RT), slides were washed in PBS and tissues were blocked (3% normal goat serum and 5% BSA in PBS) for 30 min at RT in a humidified chamber. Tissues were probed for Y705-STAT3 (1/400; no. 9145, Cell Signaling Technology), along with CD163 (1/200; MCA1853, Serotec) or CD68 clone kp1 (1/200; Dako, Carpinteria, CA) overnight at 4 °C in a humidified chamber. After washing in PBS, tissues were incubated with DyLight 594-conjugated goat anti-rabbit (Novus, Littleton, CO) and AlexaFluor-488 goat anti-mouse secondary antibodies (1/500) for 1 h at RT. Slides were again washed in PBS, mounted using SlowFade Gold with DAPI (Invitrogen) and imaged using UPlanAPO × 100, numerical aperture=1.35 oil immersion objective on an Olympus Bx51 microscope (Center Valley, PA) equipped with a SPOT RT Slider digital camera (Sterling Heights, MI).

### Western blot analysis

Whole-cell lysate was extracted from mouse peritoneal macrophage using RIPA buffer (50 mM Tris (pH=7.4), 150 mM NaCl, 1% EDTA, 1% NP-40 and 0.1% SDS, all purchased from Sigma-Aldrich) with 100 × phosphatase and proteinase inhibitor (Thermo Fisher Scientific, Waltham, MA). An amount of 20 μg lysate was resolved using NuPage gradient gels (Life Technologies) and transferred to nitrocellulose membranes (Whatman) according to manufacturer's instructions. Membranes were blocked in 2% non-fat milk (Bio-Rad) for 30 min at RT and then incubated with antibodies against STAT3 (1/1,000, no. 9139, Cell Signaling Technology), STAT3α (1/1,000; no. 8768, Cell Signaling Technology), Y705-STAT3 (1/2,000, no. 9145 or 1/1,000, no. 9138, Cell Signaling Technology), P65 (1/1,000; no. 8242, Cell Signaling Technology), phospho-P65 (1/1,000; no. 3033, Cell Signaling Technology), IKKβ (1/1,000; no. 2684, Cell Signaling Technology), phospho-IκB (1/1,000; no. 9246, Cell Signaling Technology), phospho-IκB (1/1,000, no. 9242, Cell Signaling Technology), IKKβ (1/1,000; no. 2684, Cell Signaling Technology), Arg1 (1/1,000; no. 9819, Cell Signaling Technology) SOCS3 (1/1,000; no. 2923, Cell Signaling Technology), histone deacetylase (HDAC) (1/1,000; no. 5113, Cell Signaling Technology), β-actin (1/10,000; A2228, Sigma-Aldrich) and GAPDH (1/5,000; SAB3500247, Sigma-Aldrich) in the Odyssey Blocking Buffer (Li-Cor Biosciences, Omaha, NE) with 0.01% Tween-20 (Sigma-Aldrich) at 4 °C overnight. Blots were then washed in PBST (PBS with 0.1% Tween-20) and incubated with secondary antibody conjugated to IRDye 800CW or 680CW fluorophore (1/20,000, Li-Cor Biosciences). Protein bands were detected and acquired for further analysis using Image Studio (Li-Cor Biosciences). Blot images have been cropped for presentation. Full-size blots are presented in Supplementary Figs 7 and 8. Total band intensities were first normalized to β-actin, GAPDH or HDAC, and the ratio between phosphorylated protein and total protein was used to determine the level of signalling.

### Flow cytometry

Peritoneal macrophages from young (<3 months) and old (>18 months) mice were isolated and cultured as described previously. Macrophages were blocked with mouse BD Fc Block (BD Pharmingen, San Diego, CA) before double staining with PE-Cy7-conjugated anti-mouse F4/80 (1/100; 25–4,801, clone:BM8, eBiosciences) and anti-mouse IL10R1-PE (1/100; CD210, no. 559914, clone 1B1.3a, BD Biosciences, San Jose, CA) or rat IgG1,κ-PE isotype control (1/100; no. 554685, clone:R3–34, BD Biosciences). Acquisition was carried out using LSR-II cytometer (BD Biosciences), and the data were analysed using FlowJo Software (version 7.6.2).

### Laser-induced CNV in mice

The rupture of Bruch's membrane with an Argon laser was used to induce CNV in mice as described previously^12^,^14^,^26^,^65^,^66^. All mice used in this study were female and aged between 8 and 16 weeks of age. Briefly, mice were anaesthetized by injecting ketamine hydrochloride (100 mg kg^−1^) and xylazine (13.4 mg kg^−1^) i.p., and their pupils were dilated with 1% tropicamide. Using an argon green laser, three to four laser burns were placed around the optic nerve (0.1 s, 100 μm and 700–800 mW, Phoenix Laboratory). Seven days after injury, mice were anaesthetized and perfused with 100 μl of 50 mg ml^−1^ fluorescein isothiocyanate-labelled dextran (MW 2,000,000) via the femoral vein. Eyes were enucleated and fixed immediately in 4% paraformaldehyde for 1 h. Eyes were then washed with PBS and the choroid was flat mounted onto a glass slide. The choroidal flat mount was either processed for immunohistochemical analysis or mounted directly with DAPI-containing mounting media for further imaging and acquisition using Zeiss LSM510 confocal microscopy. Z-stack images of CNV spots were then processed in ImageJ (National Institutes of Health) to create pseudo-volumetric two-dimensional images, and pixel intensity was quantified using Metamorph software analysis (Sunnyvale, CA).

For anti-IL10R1 antibody-mediated rescue, mice were injected intravitreally with either 50 μg of either isotype control or anti-IL10R1 antibodies (Serotec). Mice were treated the day of laser injury and 2 days after CNV induction.

For adoptive transfer of primary macrophage study, peritoneal macrophages elicited by thioglycollate in either Stat3 (WT) or *Stat3*^*F/F*^*;LysM*^*Cre*^ were harvested and allowed to recover in cell culture environment for 48 h. Cells were then scraped, counted and adjusted to 10 × 10^6^ cells per 100 μl. Eighteen- to 22-month-old mice were anaesthetized and injected with ∼5 × 10^5^ cells per eye immediately after laser injury was induced.

For the 5,15-DPP-mediated rescue experiment, mice received daily i.p. injection of drug (15 mg kg^−1^ body weight) or vehicle (DMSO) for 5 days until CNV was performed. Mice continued to receive daily i.p. injection of same dose until the day before killing for perfusion and choroidal flat mount processing.

### Statistics

Statistical significance was analysed by two-tailed Student's unpaired *t*-test and analysis of variance with Tukey Kramer post test with the use of GraphPad Prism Software (version 5.0). Statistical significance was defined as a *P* value <0.05.

## Additional information

**How to cite this article:** Nakamura, R. *et al.* IL10-driven STAT3 signalling in senescent macrophages promotes pathological eye angiogenesis. *Nat. Commun.* 6:7847 doi: 10.1038/ncomms8847 (2015).

## Supplementary Material

Supplementary Information Supplementary Figures 1-8
