# Supplementary material for: IL10-driven STAT3 signalling in senescent macrophages promotes pathological eye angiogenesis
Source: Nat Commun. 2015 Aug 11;6:7847. doi: 10.1038/ncomms8847 (PMC4918330; doi:10.1038/ncomms8847)
Supplement: Supplementary Information — Supplementary Figures 1-8 [file ncomms8847-s1.pdf]

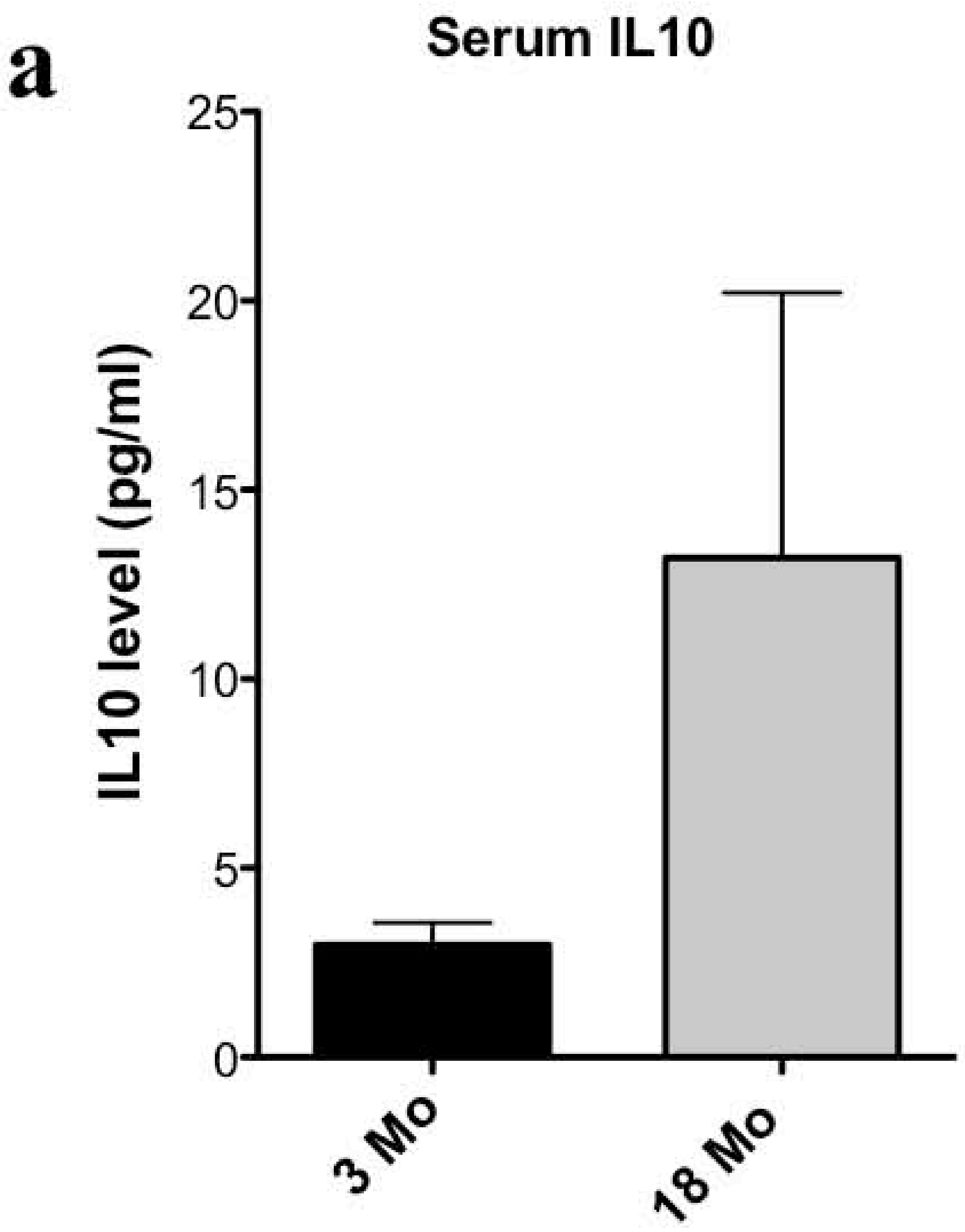

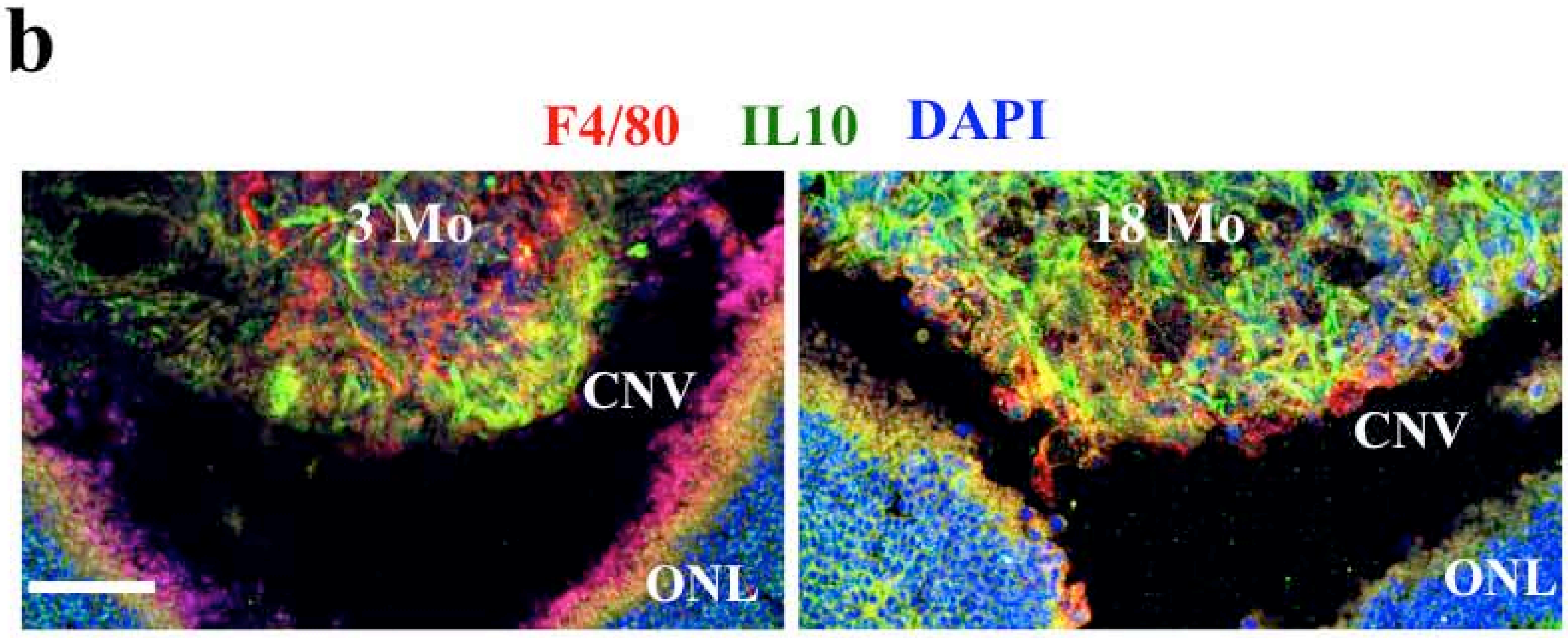

Supplementary Figure. 1. IL10 production is increased in serum and CNV complexes of old mice. (**a**) Serum level of IL10 was measured by ELISA in 3 month- (3 Mo, n=8) and 18 month-old (18 Mo, n=9) mice. (**b**) Choroidal neovascularization (CNV) was induced in 3 Mo (left panel) and 18 Mo (right panel) mice. After 7 days, eyes were cross-sectioned and stained for F4/80 (macrophage marker), IL10 and Dapi (nuclei). Scale bar = 100 μm. ONL, outer nuclear layer.

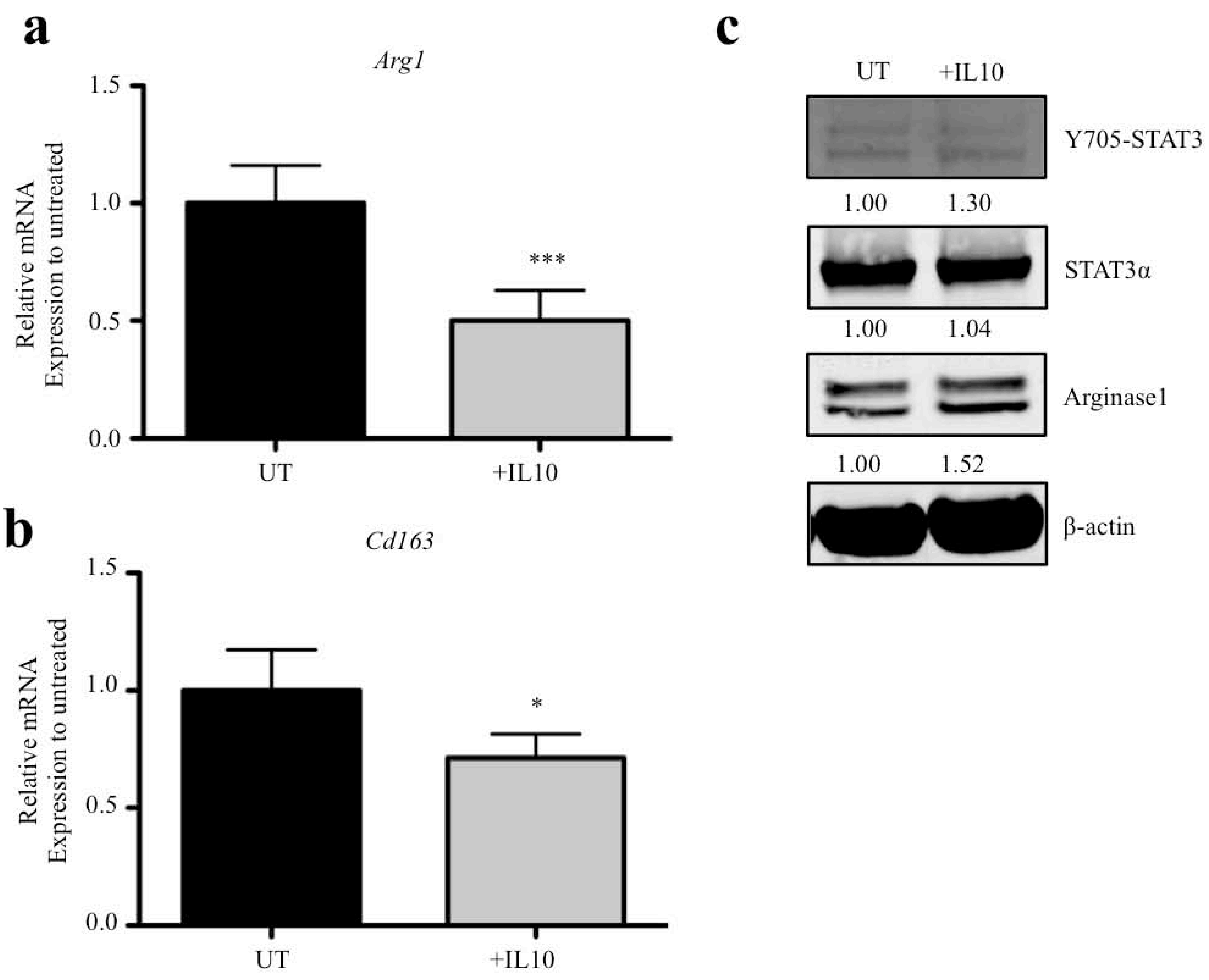

Supplementary Figure. 2. *Il10-r2*$^{-/-}$ macrophages are resistant to M2 polarization after IL10 treatment. Peritoneal macrophages from *IL10r2*$^{-/-}$ mice were treated with IL10 for 18 hours. (**a**) *Arg1* and (**b**) *Cd163* mRNA expression were measured by quantitative real-time PCR. Values are expressed as mean + SEM relative to untreated macrophage. At least three biological replicates were analyzed. (Student unpaired t test; ***p < 0.001, **p < 0.01). (**c**) Representative immunoblot for Y705-STAT3, STAT3α, Arginase1, and β–actin after 18 hours of treatment with IL10. Values underneath the blot indicate expression relative to β–actin.

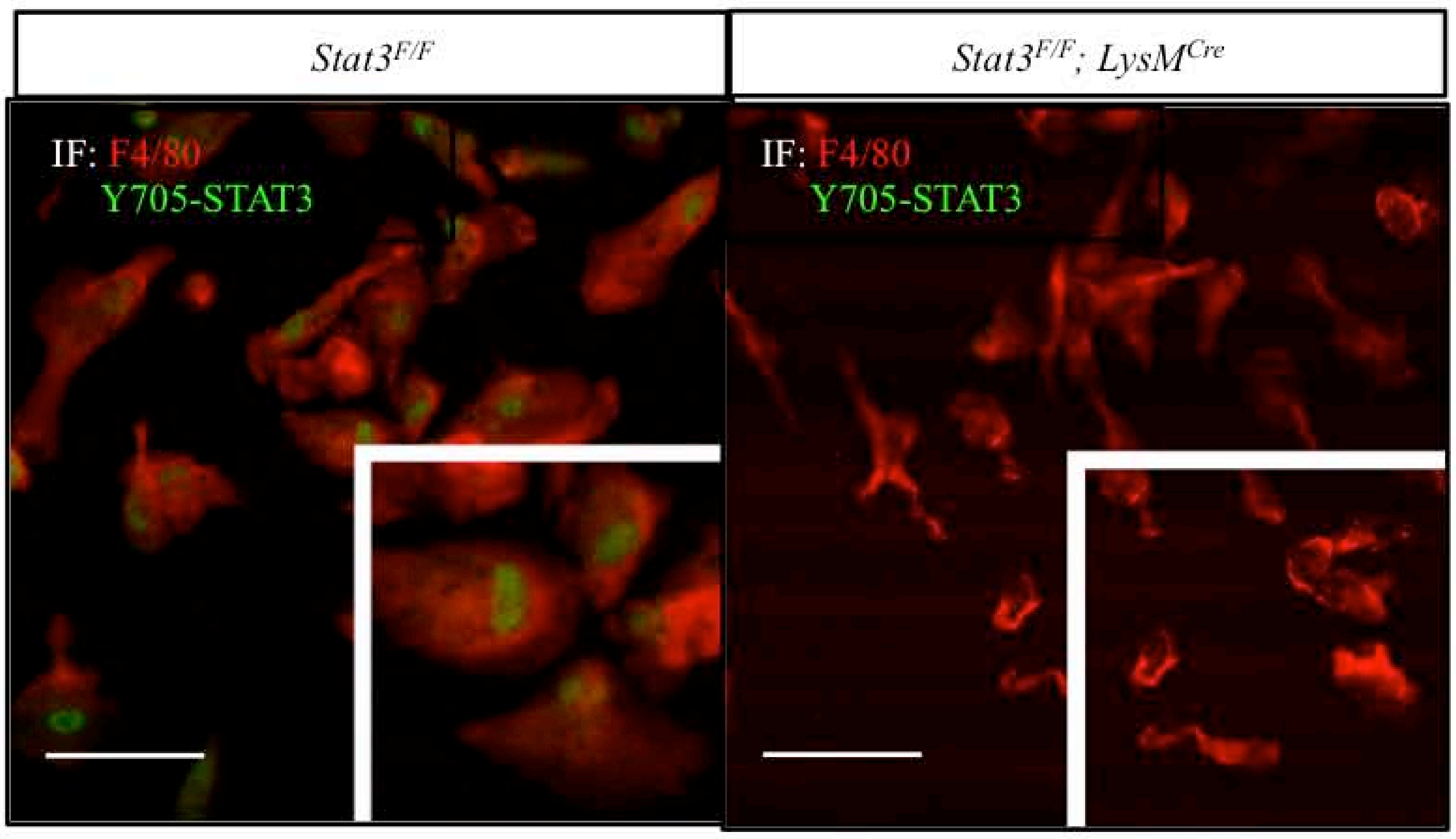

Supplementary Figure. 3. Stat3$^{F/F}$; LysM^{Cre} macrophages fails to respond to IL10 treatment through STAT3 signaling. Immunohistochemical image of phospho-STAT3 (green) after IL10 treatment in *STAT3$^{F/F}$;LysM^{Cre}* and littermate controls (*STAT3$^{F/F}$*). F4/80 (red) is used as a macrophage marker protein. *STAT3$^{F/F}$;LysM^{Cre}* macrophages do not respond to IL10 cytokine via Stat3 signaling. Representative images of staining from 3 independent experiments are shown. Scale bar = 50 µm.

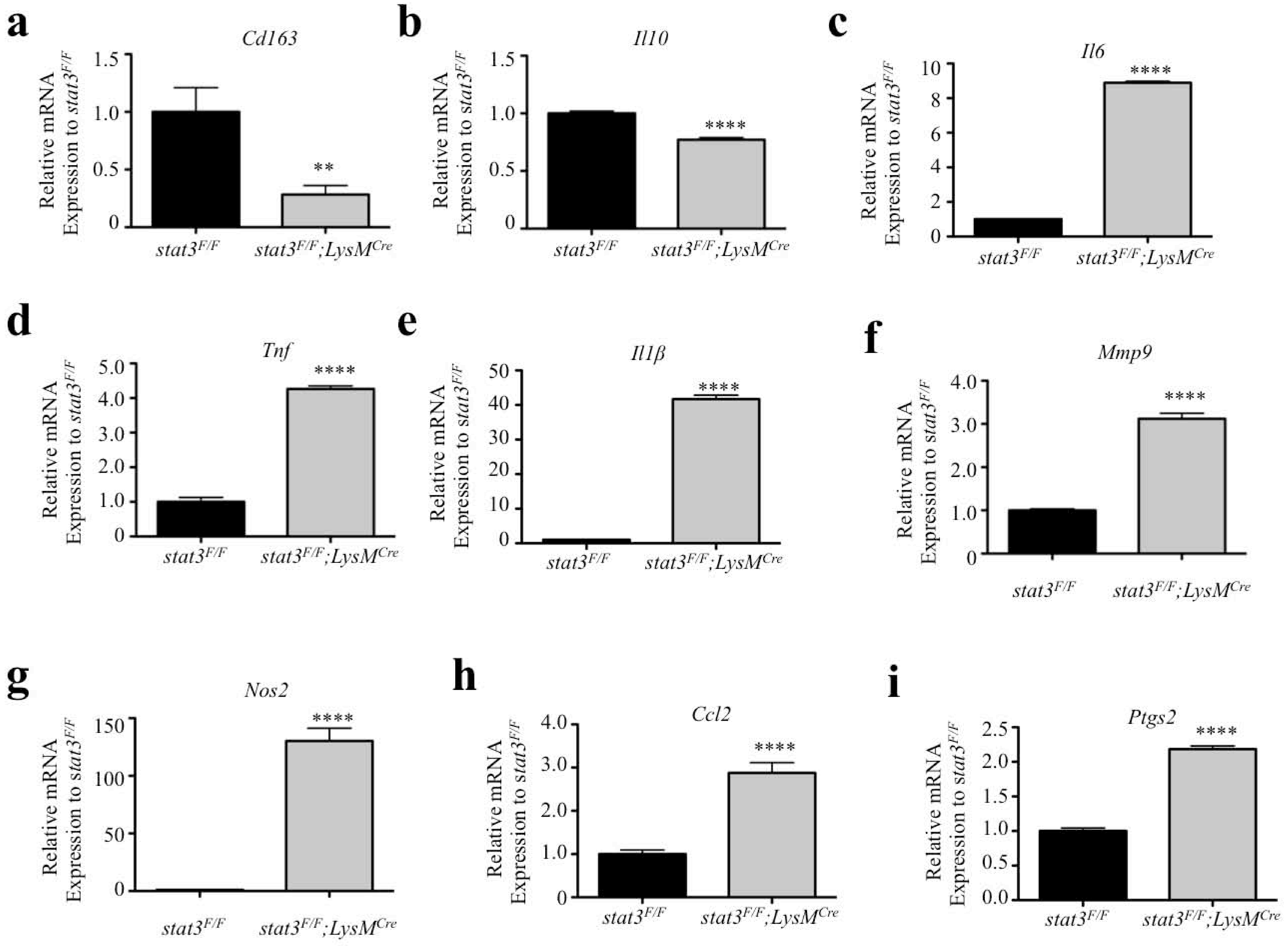

Supplementary Fig. 4. Baseline polarization of Stat3$^{F/F}$; LysM^{Cre} macrophage. Baseline macrophage polarization was determined using quantitative real-time qPCR. M2 markers (**a**) *Cd163* and (**b**) *Il10* were significantly down regulated in *STAT3$^{F/F}$;LysM^{Cre}* while M1 markers (**c**) *Il6*, (**d**) *Tnf*, (**e**) *Il1β*, (**f**) *Mmp9*, (**g**) *Nos2*, (**h**) *Ccl2*, and (**i**) *Ptgs2* were elevated. Values are expressed as mean + SEM relative to *STAT3$^{F/F}$*. (n > 5 biological replicate per group, unpaired t test; * $p < 0.05$, ** $p < 0.01$, ***$p < 0.001$ compared to *STAT3$^{F/F}$* macrophage).

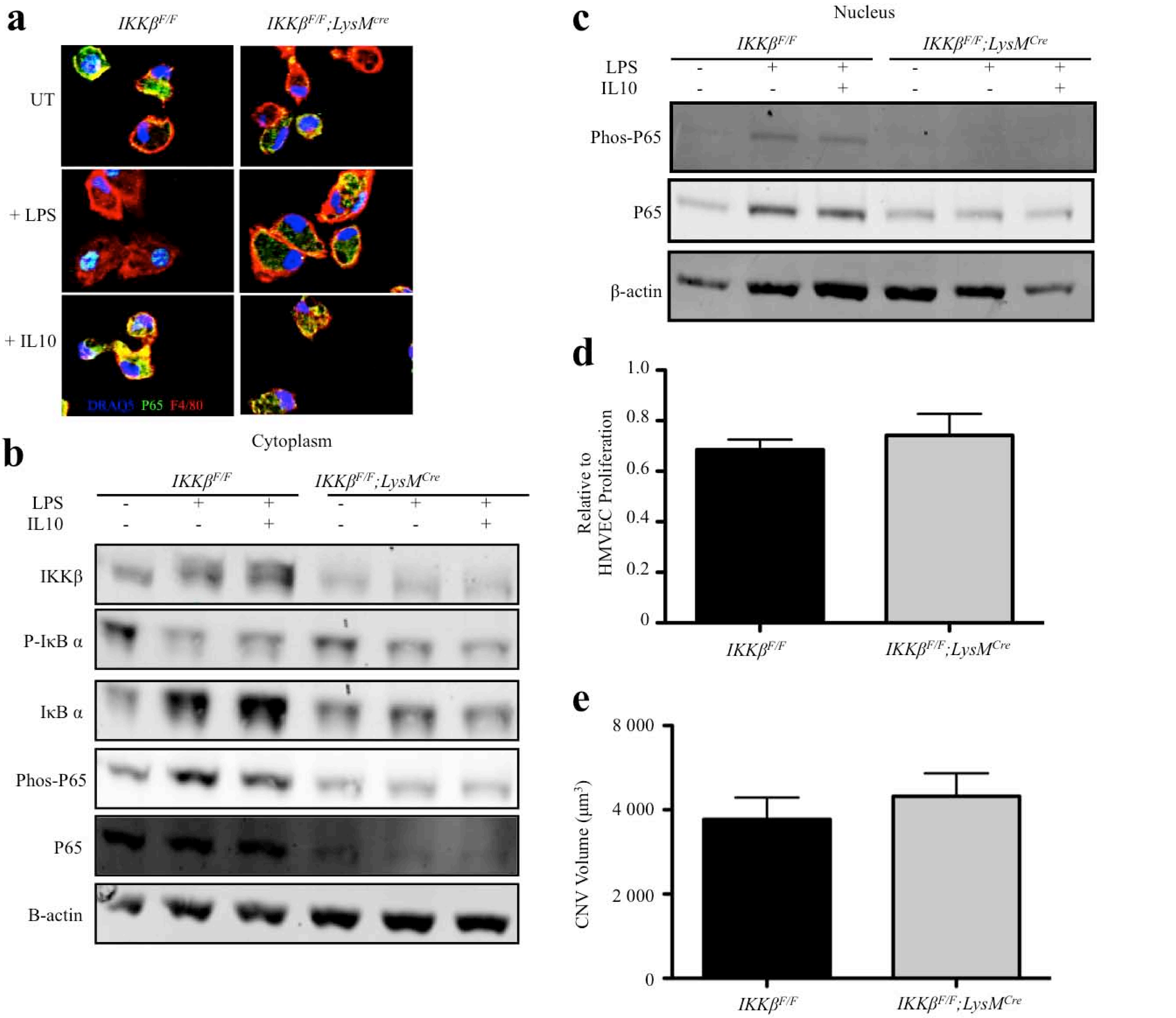

Supplementary Figure. 5. NF-kB pathway in macrophages is not involved in regulation of angiogenesis *in vivo* and *in vitro*. (**a**) Peritoneal macrophages isolated from IKKβ$^{F/F}$;LysM^{cre} or IKKβ$^{F/F}$ control mice were treated with LPS (100 ng/ml) or IL10 (100 ng/ml) for 18 hr. Immunohisochemical analysis was performed to visualize the NF-κB signaling pathway by tracing nuclear P65 expression after treatment. Representative images of the staining are shown. F4/80 and DRAQ5 were used to counterstain for macrophage and nuclei, respectively. Immunoblot for IKKβ, phospho-IKBα, IKBα, phospho-P65 and P65 subunit expression in (**b**) cytoplasmic and (**c**) nuclear lysate of IKKβ$^{F/F}$;LysM^{cre} macrophage and IKKβ$^{F/F}$ control. β–actin was used for loading control. Representative blots from 3 independent experiments are shown. (**d**) HMVEC proliferation assay demonstrates that IKKβ$^{F/F}$;LysM^{cre} does not affect HMVEC proliferation compared to littermate IKKβ$^{F/F}$ control. N = 20 co-cultures for each genotype. Student's unpaired *t* test. p value is not significant. (**e**) Laser induced choroidal neovascularization performed in IKKβ$^{F/F}$;LysM^{cre} and IKKβ$^{F/F}$ control. Mice were perfused with FITC labeled dextran to visualize CNV and to quantify CNV volume. N > 10 individual mice for each genotype. Student's unpaired *t* test. p value is not significant.

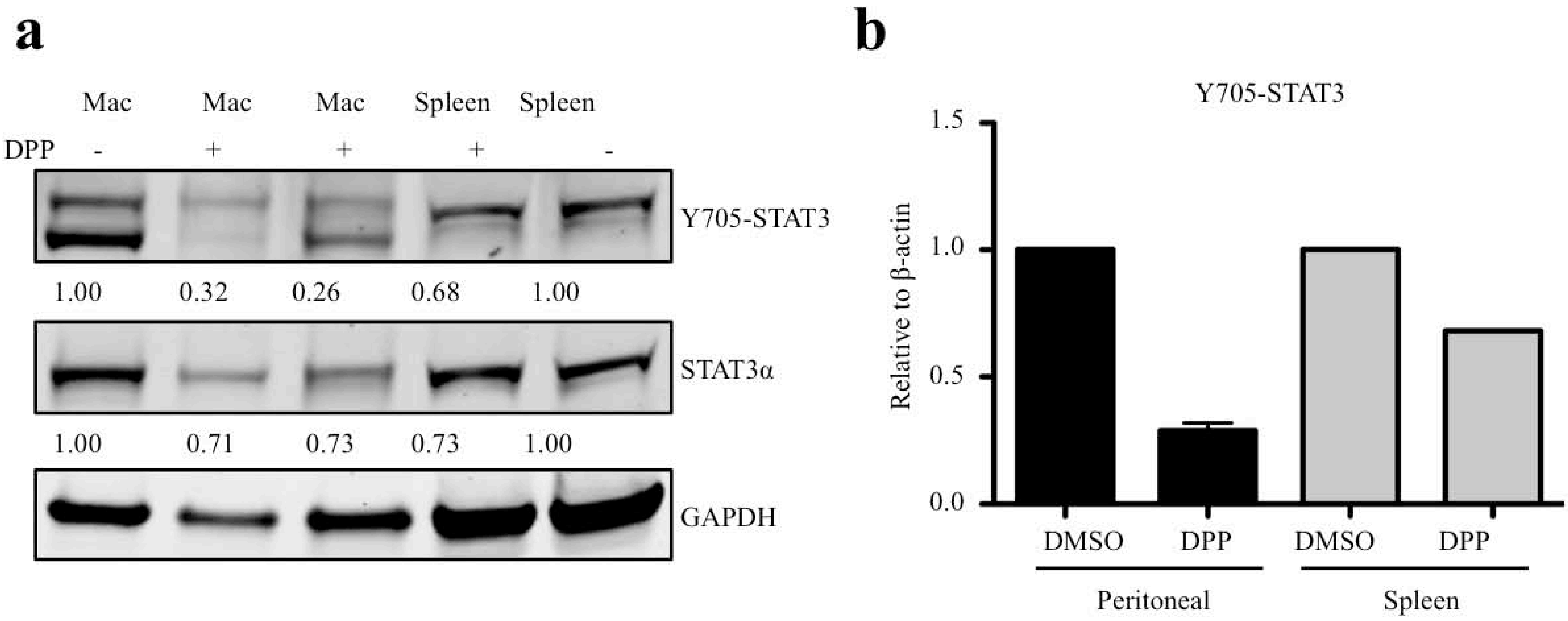

Supplementary Figure. 6. 5,15-DPP inhibits STAT3 activation *in vivo*.
(**a**) 18 month old female mice received daily 5,15-DPP (15mg/kg) or DMSO i.p. injections for 5 days before CNV injury and continued to receive injection until time for analysis. On the day of perfusion with FITC-labeled dextran peritoneal macrophages and the spleen were isolated. Whole cell lysate from macrophages and spleen were subjected to Western blot analysis for Y705-STAT3, STAT3α and GAPDH. (**b**) Band intensity was measured and normalized to untreated (DPP (-)) samples.

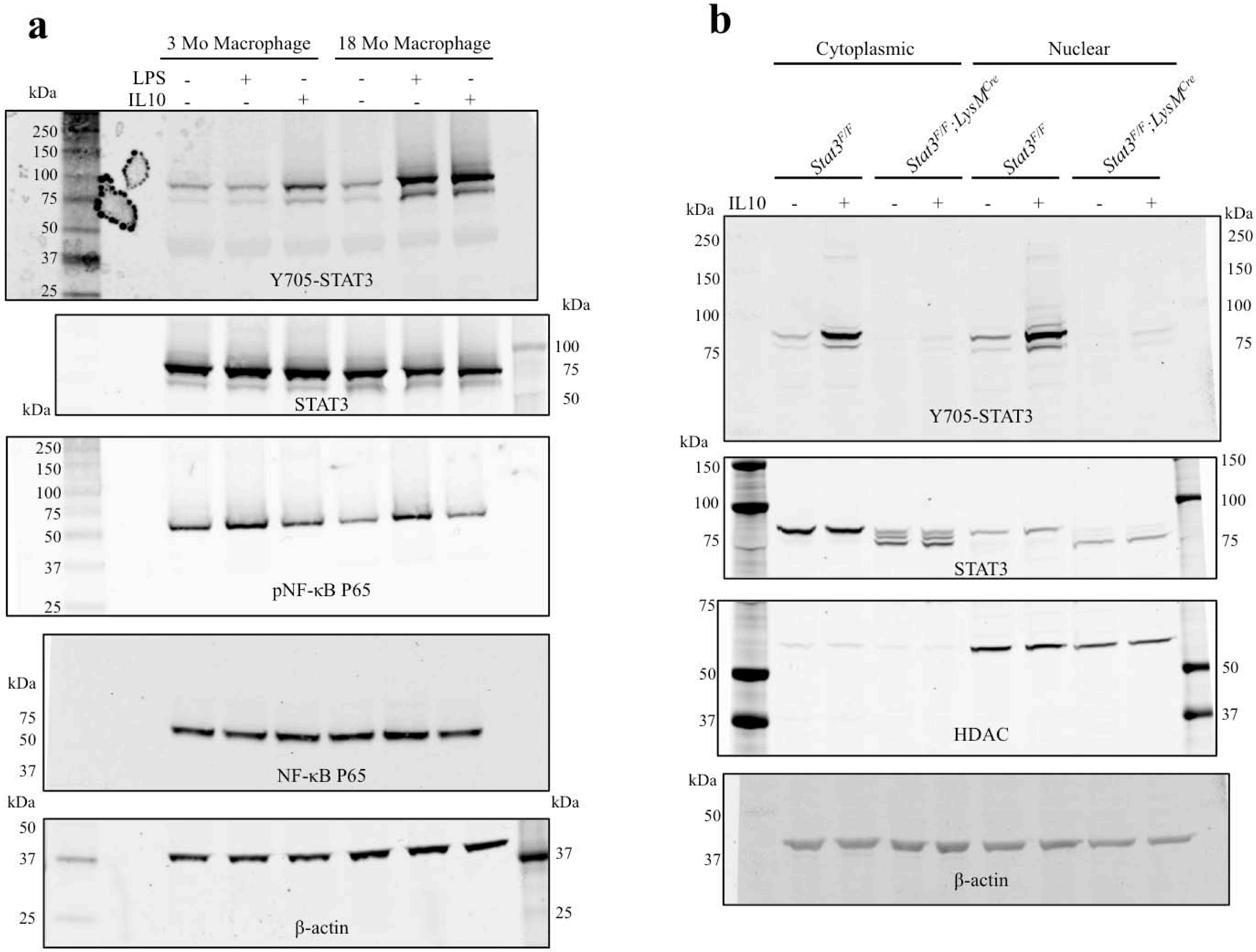

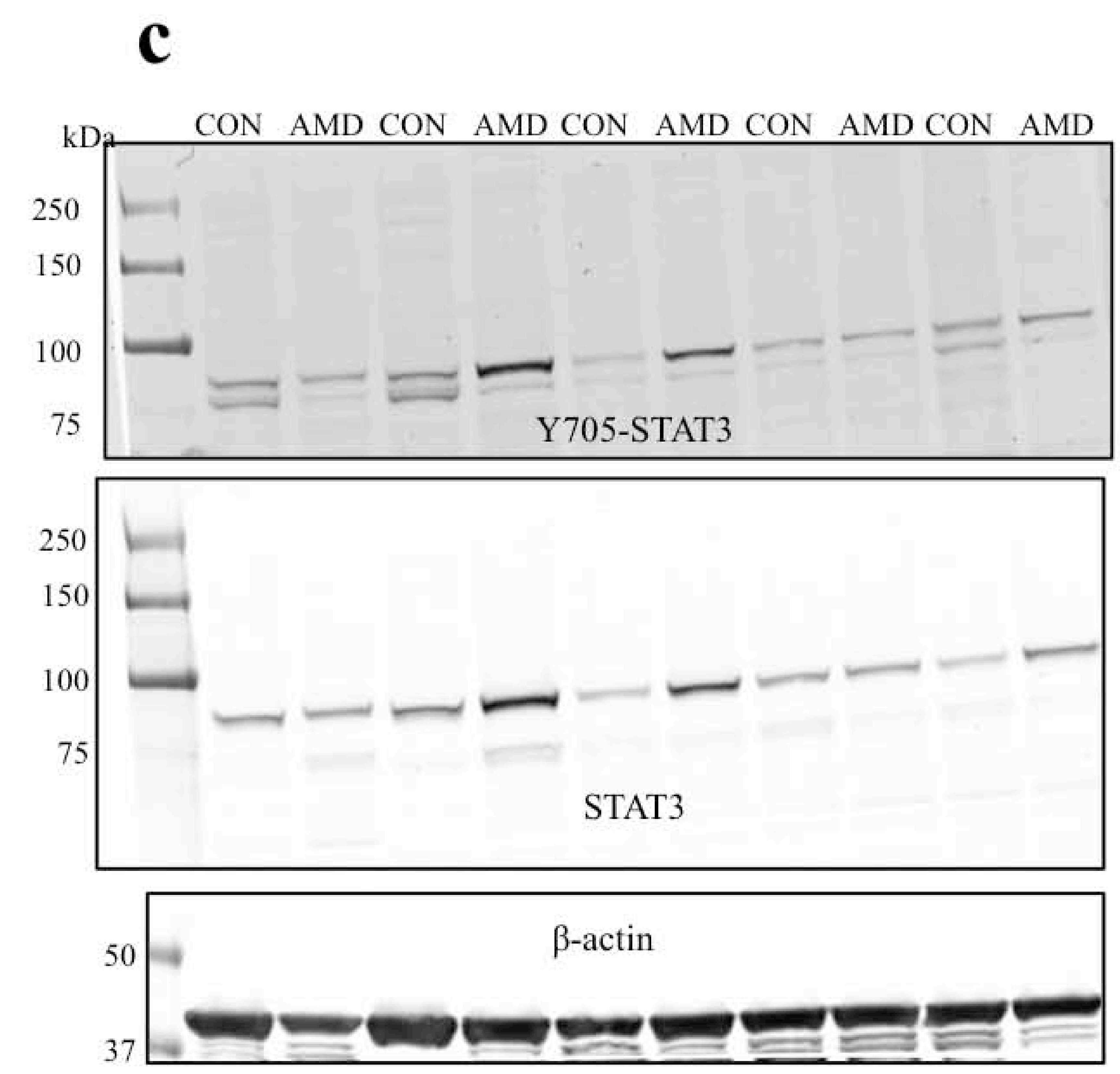

Supplementary Figure. 7. Full size blot images of sections presented in the manuscript.
Full blots of sections shown in Figure 1d (**a**), Figure 3a (**b**) and Figure 7a (**c**).

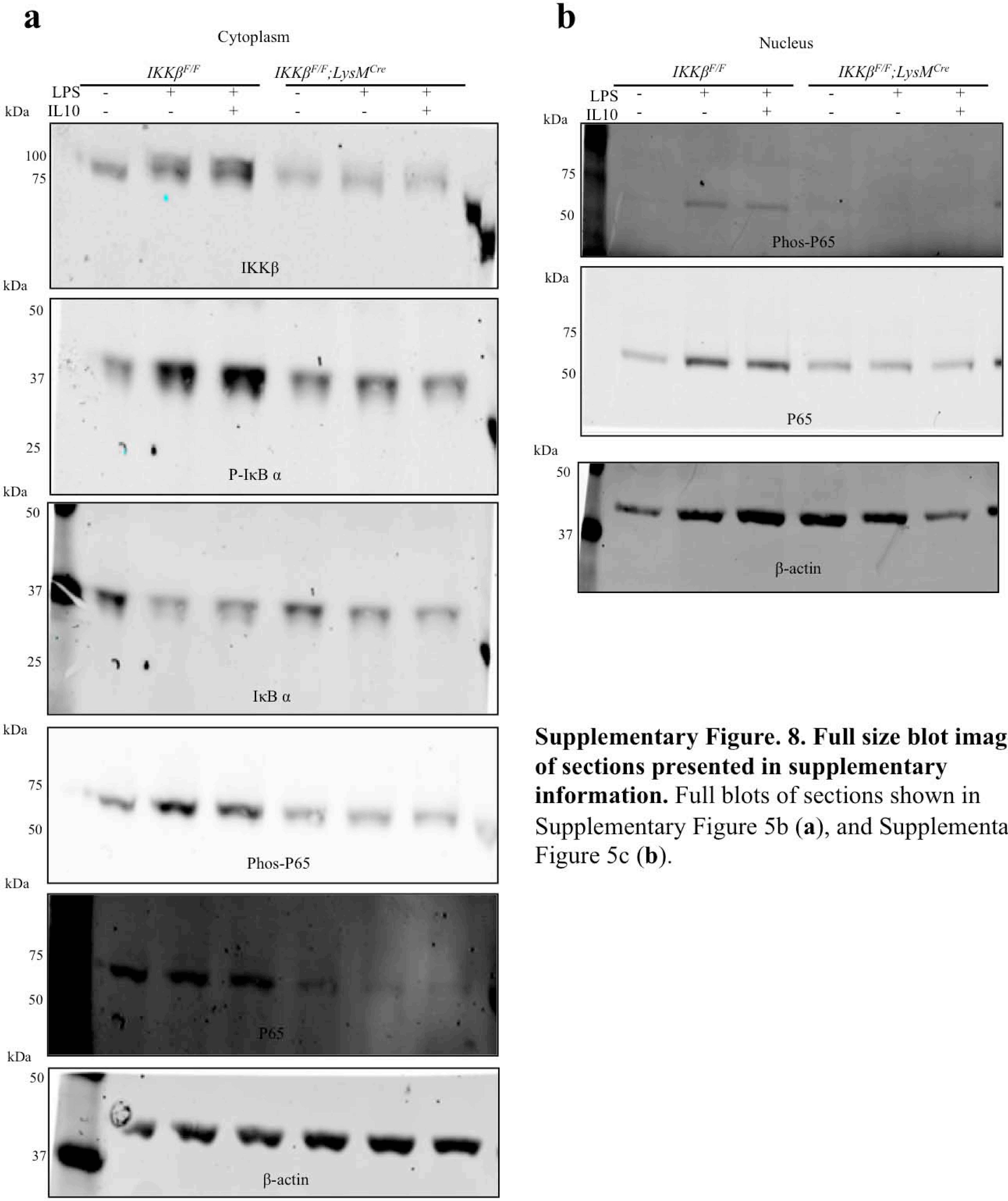

Supplementary Figure. 8. Full size blot images of sections presented in supplementary information. Full blots of sections shown in Supplementary Figure 5b (**a**), and Supplementary Figure 5c (**b**).
